# An updated overview of alkaloids for the prevention and treatment of metabolic dysfunction-associated steatotic liver disease

**DOI:** 10.3389/fphar.2026.1877634

**Published:** 2026-07-29

**Authors:** Jinguo Wang, Ying Zhang, Shuo Sun, Lanyan Hu, Dong Yang

**Affiliations:** 1 Clinical Laboratory, The 921 Hospital of Joint Logistic Support Force of PLA (The Second Affiliated Hospital of Hunan Normal University), Changsha, China; 2 Blood Transfusion Department, The 921 Hospital of Joint Logistic Support Force of PLA (The Second Affiliated Hospital of Hunan Normal University), Changsha, China

**Keywords:** alkaloids, metabolic dysfunction-associated steatotic liver disease, multi-target, natural product, pathogenesis, prevention, treatment

## Abstract

Metabolic dysfunction-associated steatotic liver disease (MASLD) has emerged as an increasingly formidable global public health challenge, characterized by a complex pathogenesis and a persistent scarcity of reliable and effective therapeutic interventions. In recent years, the multiple-hit hypothesis has provided novel mechanistic insights into the pathogenesis and progression of MASLD, paving the way for identifying viable therapeutic strategies. Due to their characteristic multi-target and multi-pathway regulatory profiles, natural products align closely with the core tenets of the multiple-hit hypothesis, demonstrating unique advantages and promising prospects in the research and development of MASLD therapeutics; consequently, they have become a prominent focal point in this field. This article provides a systematic review of the latest advances in the core pathogenesis of MASLD, summarizes key natural products with potential interventional efficacy, and highlights the critical targets, core signaling pathways, and therapeutic value of alkaloid compounds. This review aims to establish a theoretical framework and provide a scientific basis for developing innovative drugs to prevent and treat MASLD.

## 1 Introduction

In June 2023, aiming to more accurately reflect the underlying lipid metabolic issues, the American Association for the Study of Liver Diseases (AASLD) and the European Association for the Study of the Liver (EASL) reached a consensus to reclassify nonalcoholic fatty liver disease (NAFLD) and nonalcoholic steatohepatitis (NASH) as metabolic dysfunction-associated steatotic liver disease (MASLD) and metabolic dysfunction-associated steatohepatitis (MASH) (Zhang C.-Y. et al., 2025).

MASLD is a chronic liver disease. Its diagnosis requires that liver steatosis should involve more than 5% of liver cells, and at the same time, it should be accompanied by at least one metabolic risk factor (Ajmera et al., 2023; Israelsen et al., 2024), such as obesity, type 2 diabetes, hypertension, dyslipidemia or insulin resistance (IR). The occurrence and development of this disease are jointly influenced by multiple factors including lifestyle, dietary habits, drug exposure and genetic susceptibility. The increasing prevalence of metabolic risk factors is the main cause driving the continuous increase in the disease burden. Currently, about 30%–40% of adults worldwide suffer from this disease, highlighting its status as a significant public health challenge (Tilg et al., 2026). If no effective intervention is taken, its health impact will become increasingly severe. The disease can progress to MASH, presenting with liver cell damage, inflammation and fibrosis, and eventually may develop into liver cirrhosis and hepatocellular carcinoma (HCC), even requiring liver transplantation (Devarbhavi et al., 2023).

In recent years, there has been significant advancement in pharmacological studies on MASLD, with therapeutic approaches shifting from traditional lifestyle adjustments to precision-targeted treatments and combination therapies that target various pathophysiological pathways. However, current therapeutic approaches still face certain drawbacks, underscoring the need for a more integrated understanding of systemic pathogenic mechanisms. While many reviews have elaborated on the potential of natural products, including flavonoids, phenolic acids, and alkaloids, in treating MASLD, there remain three key drawbacks that need addressing. To start with, most current research tends to offer one-sided accounts that center on individual drug actions, such as improving lipid buildup, lowering inflammatory factors, or lessening oxidative stress. Consequently, they rarely adopt the multiple-hit hypothesis as a cohesive framework to systematically explore the causal links between these pathological events. Second, the regulatory mechanisms of protein post-translational modifications (PTMs) and the roles of diverse cell death pathways are not systematically elucidated, with most analyses confined to phenomenological descriptions. Additionally, the systematic exploration of how natural alkaloids interact with key regulatory factors in these pathways remains limited, hindering a comprehensive understanding of their therapeutic potential, and there is a notable lack of comparative studies between natural products and recently Food and Drug Administration (FDA)-approved drugs. This gap overlooks the possible clinical significance of natural alkaloids as supplementary or substitute treatment approaches.

To address this gap, this review takes an integrated pathological network perspective for the first time. We use the progression of the multiple-hit hypothesis as a central organizing principle to structure our analysis. We focus on key pathological nodes, including lipid dysregulation, IR, gut-liver axis disruption, gut microbiota dysbiosis, the AMP-activated protein kinase (AMPK) signaling pathway, and various forms of cell death. Against this backdrop, we systematically analyze the common and distinct regulatory patterns of three representative alkaloids—berberine (BER), nuciferine (NUC), and betaine (BET)—on these nodes. For instance, regarding the gut-liver axis, it has been established that BER ameliorates MASLD by activating the liver kinase B1 (LKB1)/AMPK signaling pathway and modulating the gut microbiota (e.g., by increasing the abundance of *Akkermansia*) (Tilg et al., 2022). However, a systematic comparison of the mechanisms of action of NUC and BET on the gut-liver axis is currently lacking. To address this gap, this review compares the effects of these three alkaloids on intercellular junction proteins and intestinal barrier function, thereby clarifying the intricate relationships among the gut microbiota, inflammatory pathways, and the AMPK signaling pathway. Additionally, we delve into the distinct regulatory functions of PTMs and the interplay of various cell death mechanisms. Concurrently, we evaluate the effectiveness and drawbacks of FDA-approved pharmaceuticals, highlighting the distinctive benefits of alkaloids as multi-target regulatory agents for disease prevention and management. This includes emerging approaches that leverage natural product alkaloids for disease prevention and management. Thereby providing a novel theoretical framework for precision treatment strategies based on the structural and target differences of alkaloids, and clarifies the future research directions and potential therapeutic targets, in order to update the research progress in this field.

## 2 Mechanisms of MASLD pathogenesis

**FIGURE 1:**
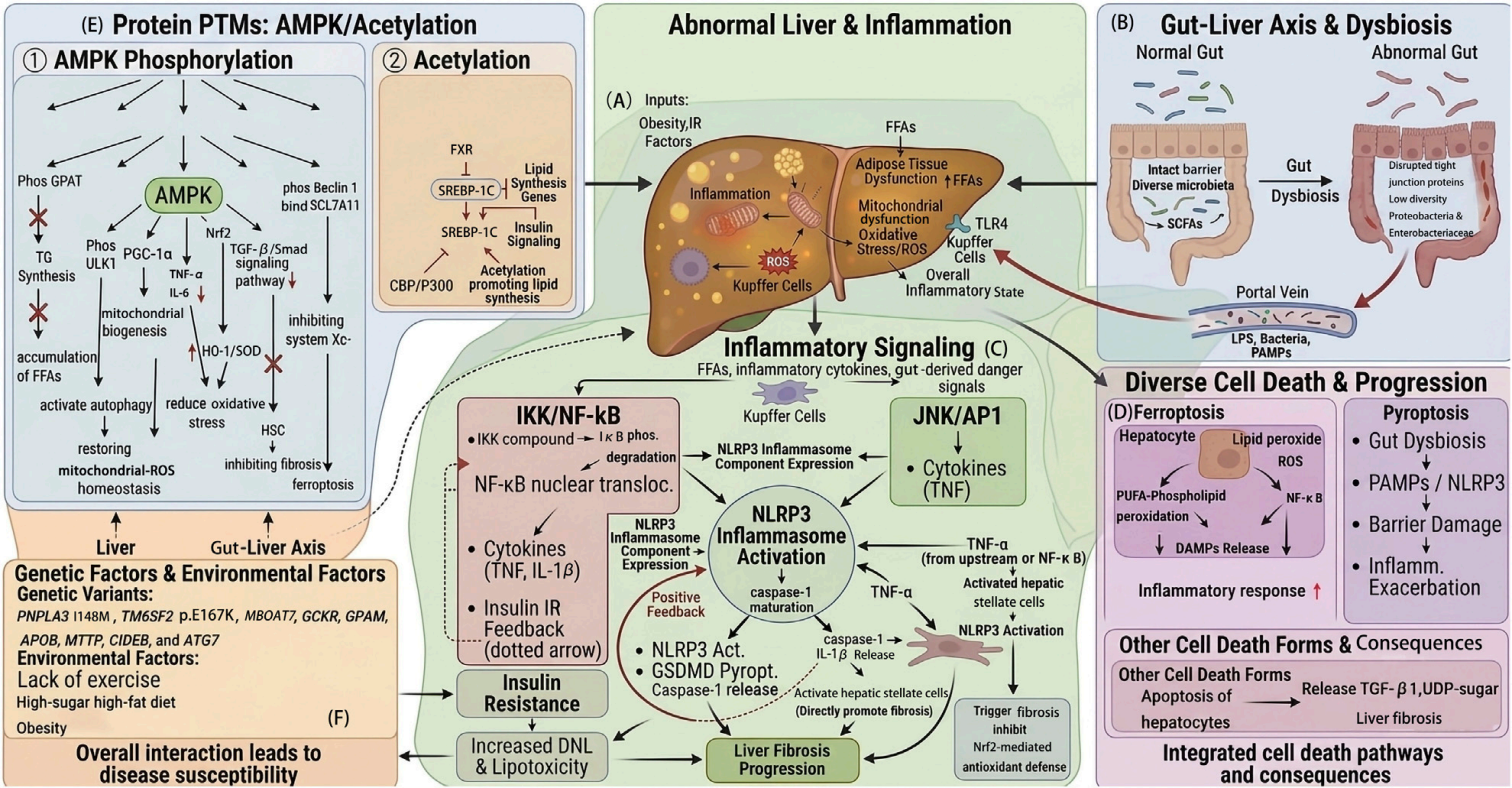
Pathogenesis of MASLD/MASH according to the multiple-hit hypothesis. As stipulated by this model, disease progression involves a complex interplay of several critical mechanisms: **(A)** Obesity and insulin resistance(IR) precipitate adipose tissue dysfunction, subsequently triggering lipid metabolism disturbances, mitochondrial impairment, and oxidative stress. **(B)** Gut dysbiosis and compromised intestinal barrier integrity activate the LPS/TLR4/NF-κB signaling cascade, which inhibits autophagy, exacerbates steatosis, and incites Kupffer cell-mediated inflammation, thereby accelerating the progression from MASLD to MASH. **(C)** Inflammatory cytokines and the NLRP3 inflammasome activate the IKK/NF-κB and JNK/AP-1 pathways, fundamentally impairing insulin sensitivity; concurrently, the NLRP3/caspase-1 axis promotes IL-1β secretion and pyroptosis, while TNF-α triggers NLRP3 activation in HSCs, suppressing Nrf2 and driving fibrogenesis. **(D)** Metabolic derangements induce ferroptosis—characterized by iron accumulation and reactive oxygen species (ROS)-mediated lipid peroxidation—which releases DAMPs to amplify hepatic inflammation. Simultaneously, dysbiosis-derived PAMPs trigger intestinal pyroptosis, further disrupting barrier integrity, whereas hepatocyte apoptosis activates HSCs via TGF-β1 and UDP-sugars to promote fibrosis. **(E)** The post-translational modifications of proteins(PTMs), such as AMPK phosphorylation and acetylation, regulate the progression of MASLD by influencing lipid metabolism, oxidative stress, mitochondrial function and inflammation, etc. **(F)** Both common and rare genetic variants heighten MASLD susceptibility through intricate interactions with environmental factors (e.g., obesity, high-fat/high-sugar diets, and IR). Notably, the PNPLA3 I148M variant specifically promotes hepatic steatosis and inflammation, serving as a primary driver in the transition from MASLD to MASH.

Metabolic-associated steatohepatitis-like disease is a multifaceted condition, characterized by the progression from systemic metabolic imbalances to subsequent cellular and molecular abnormalities. To date, however, the precise mechanisms driving its development remain incompletely understood. The conceptual framework for MASLD pathogenesis has evolved from the two-hit to the more comprehensive multiple-hit hypothesis. This updated framework provides a more nuanced explanation for the varying disease trajectories, particularly addressing why not all individuals with uncomplicated fatty liver disease progress to NASH. It also highlights the critical contributions of IR and obesity. Key classical mechanisms include the following: First, IR drives increased fatty acid synthesis and uptake in the liver, contributing to lipid buildup. This sequence creates a harmful loop with type 2 diabetes, triggering the phosphoinositide 3-kinase (PI3K)/protein kinase B (AKT)/mammalian target of rapamycin (mTOR) pathway, and consequently raising the susceptibility to inflammation, mitochondrial impairment, and liver carcinoma (Leith et al., 2024); Second, adipose tissue dysfunction releases excessive free fatty acids (FFAs) and pro-inflammatory factors, triggering endoplasmic reticulum stress and activating Kupffer cell Toll-like receptor 4 (TLR4)/nuclear factor-κB (NF-κB) pathway, thereby worsening inflammation; Third, apoptosis of hepatocytes, along with substances like transforming growth factor-β (TGF-β), triggers the activation of hepatic stellate cells (HSCs) and accelerates the development of fibrosis (Kuchay et al., 2025). Notably, recent research has identified supplementary pathogenic pathways that intersect with the multifaceted regulatory actions of alkaloids. For example, irregular PTMs may impair metabolic signaling pathways, thereby worsening fibrosis. Beyond the disruption caused by aberrant PTMs that impair metabolic signal transduction and worsen fibrosis, emerging research has highlighted the significance of ferroptosis and other cell death mechanisms in driving hepatocellular injury. Bile acids, through activation of the farnesoid X receptor (FXR) and G protein-coupled bile acid receptor 1 (TGR5), not only regulate glucose-lipid metabolism and energy balance but also interact with gut microbiota imbalance to adjust intestinal mucosal barrier function and systemic inflammatory reactions. Notably, the interplay between gut microbiota dysbiosis and bile acid signaling, along with renal sodium and water handling, collectively constitutes the complex regulatory network of the gut-liver-kidney axis (Lynggaard Elkjær et al., 2026). These newly identified mechanisms offer promising opportunities for alkaloid-based therapeutic strategies, these emerging mechanisms provide potential targets for alkaloid-based interventions. For example, BER improves metabolism by activating AMPK and modulating the microbiota, whereas NUC and BET specifically regulate barrier function, PTMs, and cell death, these processes that are central to the comparative analysis in this review. Taken together, the progression of MASLD is a dynamic process characterized by interconnected signaling networks that integrate gut microbiota, metabolic activity, inflammatory responses, and fibrogenic changes. This complexity implies that multi-targeted therapeutic approaches hold potential as a forward-looking direction (Figure 1).

### 2.1 The gut-liver axis and the intestinal microbiota

The bidirectional interaction between the intestines and the liver is known as the gut-liver axis, whose physiological importance centers on preserving systemic metabolic balance, nutrient processing, and immune stability (Anand and Mande, 2022). The gut microbiota comprises a diverse array of microorganisms, including bacteria, fungi, and viruses, which play pivotal roles in nutrient metabolism, immunomodulation, and the maintenance of the gut barrier (Wang and Zeng, 2024). Acting as an “immune filter” for gut-derived substances, the liver continuously receives microbial metabolites, dietary antigens, and pathogen-associated molecular patterns (PAMPs) via the portal vein. Under physiological conditions, hepatic innate immune cells, specifically Kupffer cells and hepatic sinusoidal endothelial cells, effectively clear these substances and induce immune tolerance, thereby maintaining gut-liver axis homeostasis. However, patients with MASLD/MASH frequently exhibit reduced microbial diversity, a depletion of beneficial symbiotic bacteria, and an overrepresentation of opportunistic pathogens, which disrupts the gut-liver axis balance by impairing the clearance function of hepatic innate immune cells like Kupffer cells and hepatic sinusoidal endothelial cells, thereby affecting the induction of immune tolerance and homeostasis maintenance. This is accompanied by elevated proportions of *Deinococcus*, *Enterobacter*, *Escherichia*, and *Bacteroides* species, with a concomitant decrease in the Firmicutes phylum. This dysbiosis directly precipitates intestinal inflammation and compromises intestinal mucosal barrier integrity (Meroni et al., 2025). Subsequent studies have demonstrated that under conditions of low oxygen and inflammatory stress, the intestinal epithelial cell cytoskeleton becomes contracted, while tight junction (Tj) proteins in the intestinal mucosa are internalized into the cytoplasm. This results in increased paracellular gaps and heightened intestinal mucosal permeability, thereby promoting the passage of bacteria and their metabolites, including lipopolysaccharide (LPS), into the liver through the portal vein. Bacteria and their byproducts like LPS then enter the liver through the portal vein, triggering the activation of pattern recognition receptors such as TLR4/MD-2 on hepatocytes, Kupffer cells, and hepatic sinusoidal endothelial cells upon their arrival at the hepatic sinusoid. This engagement leads to the recruitment of adaptor proteins myeloid differentiation primary response 88 (MyD88) or TIR-domain-containing adapter-inducing interferon-β(TRIF), followed by the activation of the IκB kinase (IKK) complex, which ultimately promotes the nuclear translocation of NF-κB (Ren et al., 2026). In liver cells, the activation of NF-κB inhibits the protective autophagy, autophagy is fundamentally a critical process for the clearance of damaged organelles and redundant lipid droplet. The suppression of its function diminishes the lipid clearance capacity of hepatocytes, leading to lipid accumulation and fatty degeneration. Meanwhile, in Kupffer cells and liver sinusoidal endothelial cells, NF-κB that has translocated into the nucleus promotes the production of inflammatory molecules including tumor necrosis factor-α (TNF-α), interleukin-1β (IL-1β) and C-C motif chemokine ligand 2(CCL2), which in turn recruit monocytes and macrophages to the liver, resulting in significant damage to liver parenchymal cells (Liu L. et al., 2022; Benedé-Ubieto et al., 2024). Consequently, a vicious cycle is established among gut microbiota imbalance, intestinal barrier damage and liver immune dysfunction, collectively driving the progression of MASLD/MASH.

### 2.2 Inflammatory pathways

Previous research has confirmed that inflammatory cytokines are crucial in the development of MASLD, disrupting insulin signaling through the activation of multiple inflammatory pathways in a positive feedback mechanism (Khan et al., 2019). Key inflammatory mediators include IL-1β, interleukin-6 (IL-6), TNF-α, C-reactive protein (CRP), and the NOD-like receptor protein 3 (NLRP3) inflammasome (Auguet et al., 2020). Notably, key inflammatory signaling pathways such as the IKK/NF-κB and c-Jun N-terminal kinase/activator protein 1 (JNK/AP-1) cascades are well-characterized in mediating the systemic response to infection and tissue damage. CRP, acting as a liver-derived, non-specific acute-phase protein (Zhang P. W. et al., 2025), can upregulate NF-κB activity, which in turn perturbs insulin signaling. The activation of the NLRP3 inflammasome constitutes a crucial juncture in the progression from simple steatosis to MASH and hepatic fibrosis; its activation leads to the maturation of caspase-1. On the one hand, caspase-1 promotes the maturation and secretion of pro-inflammatory cytokines such as IL-1β. IL-1β not only directly drives fibrogenesis but also induces NF-κB activation, thereby establishing a vicious cycle of pro-inflammatory signaling (de Carvalho Ribeiro and Szabo, 2022). On the other hand, caspase-1 cleaves gasdermin D (GSDMD) to induce hepatocyte pyroptosis and the subsequent release of inflammatory mediators (Duan et al., 2022). Furthermore, in the context of hepatic fibrosis, TNF-α can activate the NLRP3 inflammasome in hepatic stellate cells via its specific receptor, triggering fibrogenesis and suppressing nuclear factor erythroid 2-related factor 2 (Nrf2)-mediated antioxidant defenses (Ramos-Tovar and Muriel, 2020).

### 2.3 Diverse forms of cell death

Programmed cell death (PCD) is an active cellular process triggered by various biological and pathological cues, which is crucial for preserving tissue balance and supporting organismal growth (Bedoui et al., 2020). As scientific inquiry advances, the understanding of PCD has evolved to embrace various subtypes, such as necroptosis, apoptosis, pyroptosis, autophagic cell death, and ferroptosis (Griffioen and Nowak-Sliwinska, 2022). A growing body of research indicates that metabolic disruptions in MASLD act as key initiators of hepatocellular PCD, significantly contributing to the development of MASLD to MASH. Notably, ferroptosis is marked by two key features: intracellular iron accumulation and lipid peroxidation. In the context of MASLD, the primary cells affected by ferroptosis are hepatocytes. Ferroptosis typically arises from the buildup of harmful lipid peroxides and reactive Oxygen species (ROS). A key factor in this process is the presence of polyunsaturated fatty acids (PUFAs), especially when integrated into membrane phospholipids (PUFA-PLs), which act as essential substrates for lipid peroxidation. Their subsequent peroxidation compromises cell membrane integrity and generates oxidized products, thereby facilitating the release of damage-associated molecular patterns (DAMPs), such as mitochondrial DNA and oxidized lipids (Jia et al., 2021). Similarly, the activation of NF-κB can trigger the formation of sublethal necrosomes in hepatocytes, thereby promoting the release of DAMPs and exacerbating the inflammatory response (Vucur et al., 2023). Furthermore, upon gut microbiota dysbiosis, intracellular PAMPs induce intestinal epithelial cell (IEC) pyroptosis via the NLRP3 inflammasome or the caspase-4/5/11 pathway, thereby compromising gut barrier integrity and amplifying both local and systemic inflammatory responses (Wu et al., 2021). Additionally, hepatocyte apoptosis drives disease progression by releasing TGF-β1 and UDP-glucose/UDP-fructose; the former promotes extracellular matrix deposition via the TGF-β1/Smad axis, while the latter activates hepatic stellate cells via the P2Y14 receptor, synergistically culminating in hepatic fibrosis (Mederacke et al., 2022; Tang et al., 2025).

### 2.4 Protein post-translational modifications

Protein post-translational modifications (PTMs) involve the covalent addition of chemical groups to amino acid side chains or protein termini, including processes like phosphorylation, ubiquitination, and acetylation. These modifications can alter protein structure and functionality. When PTMs are not properly regulated, they can interfere with liver lipid metabolism, increase oxidative stress, impair mitochondrial activity, and affect the inflammatory response, thereby accelerating the development and progression of MASLD (Min et al., 2025). These PTMs primarily target key proteins, such as histones, membrane proteins, and secretory proteins, through which they exert their regulatory effects. A single protein can be subject to multiple PTM processes simultaneously or regulated by a specific PTM during distinct developmental periods. Within liver cells, protein acetylation exerts an impact on the development of MASLD/MASH through the control of critical molecules involved in lipid metabolism. A pivotal regulatory mechanism in this context involves the upstream bile acid receptor, FXR. When FXR is activated, it acts to repress the expression of the transcription factor sterol regulatory element-binding protein 1c (SREBP-1c), thereby decreasing the transcription of genes involved in lipid synthesis (Miyata et al., 2022). The rate-limiting enzyme for fatty acid synthesis, acetyl-CoA carboxylase (ACC), is transcriptionally regulated by SREBP-1c, and its catalytic activity can also be directly inhibited by AMPK-mediated phosphorylation at the Ser^79^ residue. Should these key pathways encounter disruptions, the body’s lipid synthesis mechanism becomes overactive, while its breakdown processes are impaired, collectively driving the advancement of pathological conditions.

As a key energy regulator, AMPK carries out diverse regulatory functions via phosphorylation-mediated signaling cascades (Smith et al., 2026). For instance, phosphorylated glycerol-3-phosphate acyltransferase (GPAT) directly suppresses triglyceride production; activated Unc-51-like autophagy activating kinase 1 (ULK1) enhances autophagic clearance of impaired mitochondria, while simultaneously stimulating peroxisome proliferator-activated receptor gamma coactivator 1α (PGC1α)-mediated mitochondrial biogenesis, thus interrupting the pathological loop of reactive ROS overaccumulation and mitochondrial dysfunction (Tolis et al., 2026). Additionally, AMPK inhibits the NF-κB pathway to decrease the secretion of pro-inflammatory cytokines like TNF-α and IL-6, and promotes the nuclear translocation of Nrf2 to upregulate cytoprotective enzymes such as heme oxygenase-1 (HO-1) and superoxide dismutase (SOD), thereby alleviating oxidative damage. Beyond its established roles in energy metabolism, AMPK also modulates cellular stress responses through multiple mechanisms, such as inhibiting the TGF-β/Smad pathway to suppress HSC activation and block hepatic fibrosis, while its role in regulating ferroptosis often depends on the specific context (Zhang et al., 2024). However, its impact on ferroptosis, a form of regulated cell death driven by lipid peroxidation, is context-dependent and can act bidirectionally. In many cases, AMPK activation suppresses ferroptosis: for example, under energy stress, it lowers polyunsaturated fatty acid production through ACC phosphorylation (Lee et al., 2020); in diabetic cardiomyopathy and acute kidney injury models, it mitigates ferroptosis via the Nrf2/HO-1 and glutathione peroxidase 4 (GPX4) pathway (Rong et al., 2026); and in M2 microglia-derived exosomes and skeletal muscle ischemia-reperfusion scenarios, it enhances mitophagy through the AMPK/ULK1 axis to achieve protective effects (Li J. et al., 2025). However, under certain circumstances, AMPK activation may also trigger ferroptosis: for example, apigenin and fucoxanthin respectively induce tumor cell ferroptosis through the AMPK and AMPK/Nrf2/heme oxygenase 1 (HMOX1) pathways (Wang Y. Z. et al., 2025). Besides, AMPK can phosphorylate Beclin1 to suppress the function of system Xc- (Cheng et al., 2024). Furthermore, the AMPK agonist A769662 has been shown to directly suppress SLC7A11-dependent cystine uptake in an AMPK-independent manner, thereby triggering ferroptosis. This finding indicates that the interplay between pharmaceutical compounds and AMPK might also result in non-canonical biological effects (Misono et al., 2026). Currently, whether AMPK regulates hepatocyte ferroptosis within MASH remains to be fully elucidated, necessitating comprehensive evaluations that integrate factors such as cell type, metabolic status, and interventional modalities.

### 2.5 Genetic and environmental factors

Growing evidence suggests that the onset and progression of MASLD/MASH are mainly governed by genetic predispositions, external circumstances, and the intricate interplay between these two elements. When examining the genetic aspects at play, numerous risk factors have been pinpointed, ranging from common variants such as *PNPLA3* I148M, *TM6SF2* p.E167K, *MBOAT7*, *GCKR*, and *GPAM*, to rarer ones like *APOB*, *MTTP*, *CIDEB*, and *ATG7* (Haas et al., 2021; Jamialahmadi et al., 2021; Baselli et al., 2022). Initial genetic studies identified the *PNPLA3* I148M variant as a key genetic determinant in the global prevalence of MASLD (Younossi et al., 2025). When individuals are subjected to pathogenic elements such as obesity, excessive consumption of high-sugar and high-fat diets, and IR, the detrimental effects of this genetic change become more significant, serving as a primary catalyst for the onset of MASLD. Beyond genetic and metabolic influences, environmental elements play a crucial role in the development of MASLD. The joint Clinical Practice Guidelines on the Management of MASLD, issued by EASL–EASD–EASO, strongly advocate the prevention of MASLD/MASH through the adoption of a healthy diet and a minimum of 150 min of physical activity per week (European Association for the Study of the Liver et al., 2024). Consequently, lifestyle intervention has historically been, and remains, the cornerstone of disease management (Peverelle et al., 2025). However, its long-term effectiveness is often undermined by suboptimal patient compliance and significant variability in therapeutic outcomes. The fundamental limitation of lifestyle modification lies in its inability to precisely modulate specific pathophysiological cascades. It is worth noting that although genetic variants such as *PNPLA3* I148M are well-recognized determinants of susceptibility to MASLD, whether they also influence the therapeutic responsiveness to pharmacological interventions, particularly multi-target natural product alkaloids, remains an unresolved pivotal issue warranting dedicated investigation.

## 3 Current pharmacological interventions for MASLD

Since MASLD pathogenesis encompasses complex mechanisms such as lipid metabolism disorders and inflammatory responses, there have been notable advancements in the creation of drugs that act on specific molecular targets. Resmetirom represents the world’s first-in-class agent approved by the U.S. FDA for the treatment of MASLD/MASH. In meta-analyses, the relative risk (RR) for achieving an improvement in fibrosis of ≥1 stage without exacerbation of MASH was 1.61, which was lower than that of FGF21 analogues (RR = 2.22) and SGLT2 inhibitors (RR = 2.27), suggesting that this intervention was effective but with a relatively weak effect (Tang et al., 2026). Mediated by organic anion transporting polypeptide 1B1 (OATP1B1), this drug specifically targets hepatocytes, thereby selectively activating intrahepatic thyroid hormone receptor β (THRβ) while circumventing the activation of THRα, which is predominantly expressed in the heart and bone tissue. This mechanism facilitates the localized mimicry of the beneficial metabolic effects of thyroid hormones within the liver (Hönes et al., 2022). In preclinical studies, resmetirom has shown promise in lowering liver triglycerides (TG), fatty liver condition, inflammatory processes, scarring, plasma alanine transaminase (ALT) concentrations, cholesterol, and blood glucose levels, while also improving both the MASLD activity score and fibrotic biomarkers without altering body mass (Wang X. J. et al., 2023). Regarding applicability, the German Steatotic Liver Disease (SLD) registry study revealed that based on three criteria: liver biopsy, US expert recommendations (VCTE 10–15 kPa with platelets ≥140 × 10^9^/L, or VCTE 8–15 kPa), and FAST ≥ 0.67 combined with liver stiffness <15 kPa, the proportions of patients meeting the eligibility criteria for resmetirom therapy were 34%, 18.3%, and 6%, respectively. This underscores that the selection of diagnostic modalities significantly influences the potential target population (Messer et al., 2025). Although resmetirom has achieved milestone approval, its therapeutic application is constrained by adverse events, primarily diarrhea and nausea. These limitations on safety have forced its use to be tightly confined to adult patients with MASH and moderate-to-severe liver fibrosis, requiring careful clinical assessment before any treatment is given. However, the long-term safety and overall health consequences of this medication are still being studied.

Subcutaneous semaglutide is a glucagon-like peptide-1 (GLP-1) receptor agonist utilized with FDA approval in the management of metabolic disorders associated with MASLD. While its exact working mechanisms are not fully understood, its effectiveness is widely recognized for its ability to address obesity and diabetes-related issues. Compared to analogous agents, semaglutide achieves up to a threefold greater reduction in body weight and a 25% decrease in average energy intake. Beyond its well-documented benefits for weight management and glycemic control, recent studies have demonstrated that semaglutide also improves plasma lipid levels and reduces inflammatory markers in clinical settings (Davies et al., 2021; Rubino et al., 2022). Interim results released in April 2025 indicated that among subjects receiving semaglutide therapy, 62.3% achieved significant remission of MASH, 36.8% exhibited a reduction in hepatic fibrosis severity, and 32.7% attained both steatohepatitis resolution and hepatic fibrosis improvement. Network meta-analyses have demonstrated that the RR of GLP-1 receptor agonists (including semaglutide) for improving fibrosis (a reduction of ≥1 stage without exacerbation of MASH) is 1.51 (95% CI: 1.09–2.10) (Koh et al., 2026). However, its practical use in clinical settings is still limited by certain factors. Gastrointestinal adverse reactions, including nausea, vomiting, abdominal pain, and altered bowel habits, occur in 20%–40% of patients. Furthermore, isolated pancreatobiliary adverse events, like acute pancreatitis, cholelithiasis, and cholecystitis, have also been documented (Zacharia et al., 2025b).

Compounds that simultaneously target GLP-1, GIP, and glucagon receptors have demonstrated substantial promise for addressing MASLD/MASH. In clinical trials, the dual-acting GIP/GLP-1 receptor agonist tirzepatide and the dual GLP-1/glucagon receptor agonist survodutide have both been shown to improve steatosis, inflammation, and fibrosis (Zafer et al., 2025). Among these, the triple agonist combining GLP-1, GIP, and insulinotropic activity, retatrutide, has achieved the most notable results, with preclinical data indicating it can reduce body weight by as much as 24.2% and liver fat content by 82%, along with potential anti-tumor immune regulatory effects (Goldney et al., 2025). Meanwhile, emerging therapeutic candidates, including cotadutide and efinopegdutide as dual GLP-1R/GIPR agonists, FGF21/GLP-1/GIP triple agonists, and GLP-1R-GIPR-PPARα/γ/δ quintuple agonists, are also being explored in clinical development, offering additional possibilities for MASLD/MASH treatment (Liskiewicz et al., 2026). Compounds like FGF21 agonists, such as efruxifermin and pegozafermin, exert beneficial effects by addressing steatosis, enhancing insulin sensitivity, and demonstrating anti-inflammatory and anti-fibrotic properties (Jeong et al., 2024). Among these agents, certain compounds demonstrate superior efficacy in mitigating liver scarring, accompanied by adverse effects primarily manifesting as mild to moderate digestive system issues. This treatment has advanced to phase III clinical trials, though further validation through extensive studies remains necessary (Rose et al., 2025).

The fatty acid synthase (FASN) inhibitor denifanstat (TVB-2640) has entered late-stage clinical trials and has shown initial anti-fibrotic efficacy (Zacharia et al., 2025a). FASN inhibitors ameliorate metabolic disorders by inhibiting fatty acid synthesis; however, because MASLD involves multiple pathways, combination therapy with Peroxisome Proliferator-Activated Receptor (PPAR) or GLP-1 receptor agonists may be required (Lee et al., 2025). In contrast, ACC inhibitors block the liver’s ability to synthesize fatty acids from scratch, thereby indirectly boosting fatty acid oxidation (Liu X. H. et al., 2026; Wölfle et al., 2026). However, a meta-analysis comprising six randomized controlled trials demonstrated that ACC inhibitors failed to improve hepatic fibrosis while markedly increasing the risk of hypertriglyceridemia (OR = 10.33) (Hasanatuludhhiyah et al., 2025). Although combining PPAR or THRβ agonists can alleviate this side effect, it does not enhance the anti-fibrotic effect. Long-term safety, efficacy persistence, and drug resistance remain major challenges. Looking ahead, efforts should focus on enhancing target specificity, investigating combined therapeutic approaches, and performing extensive randomized controlled trials to validate these findings. Other drug candidates have demonstrated promising results in preclinical studies. For instance, animal experiments indicate that SGLT2 inhibitors can effectively reduce body weight, blood glucose levels, and liver fat accumulation in mice models of diabetes complicated by MASLD, with the PI3K/Akt signaling pathway being involved in these beneficial effects (Sun, 2025). Furthermore, the antioxidant vitamin E is recommended as a first-line adjuvant therapy for patients with diabetes-free MASH (Knorr et al., 2026). Additionally, interventions targeting a new inhibitor of CYP4A have also demonstrated the potential to improve lipotoxicity, oxidative stress, and fibrosis in preclinical models (Lee et al., 2026).

Although these agents operate via different mechanisms and target different conditions, they all face a similar problem in treating current MASLD: either their efficacy still needs long-term observation to confirm, or their application in clinics is limited by significant side effects that impact the digestive system or pancreatobiliary functions. Thus, there remains an urgent need to develop new drugs that offer significant therapeutic benefits, fewer adverse effects, enhanced safety, and more holistic treatment strategies. Against this backdrop, natural products, characterized by their relatively gentle regulatory effects, have come to light as potential supplementary or alternative approaches for the prevention and control of MASLD. Recently, significant advancements have been achieved in assessing the potential of natural products in addressing MASLD. These natural derivatives, which are rich in a wide variety of phytochemicals like flavonoids, phenolic acids, saponins, polysaccharides, terpenoids, and alkaloids, demonstrate distinct therapeutic potential by synergistically targeting multiple key drivers of MASLD across different levels, such as lipid overaccumulation, oxidative stress, inflammatory responses, and imbalanced gut microbiota composition. Such combined effects set them apart noticeably from traditional single-action chemical medications.

## 4 Natural product alkaloids and therapeutic targets for MASLD

**FIGURE 2:**
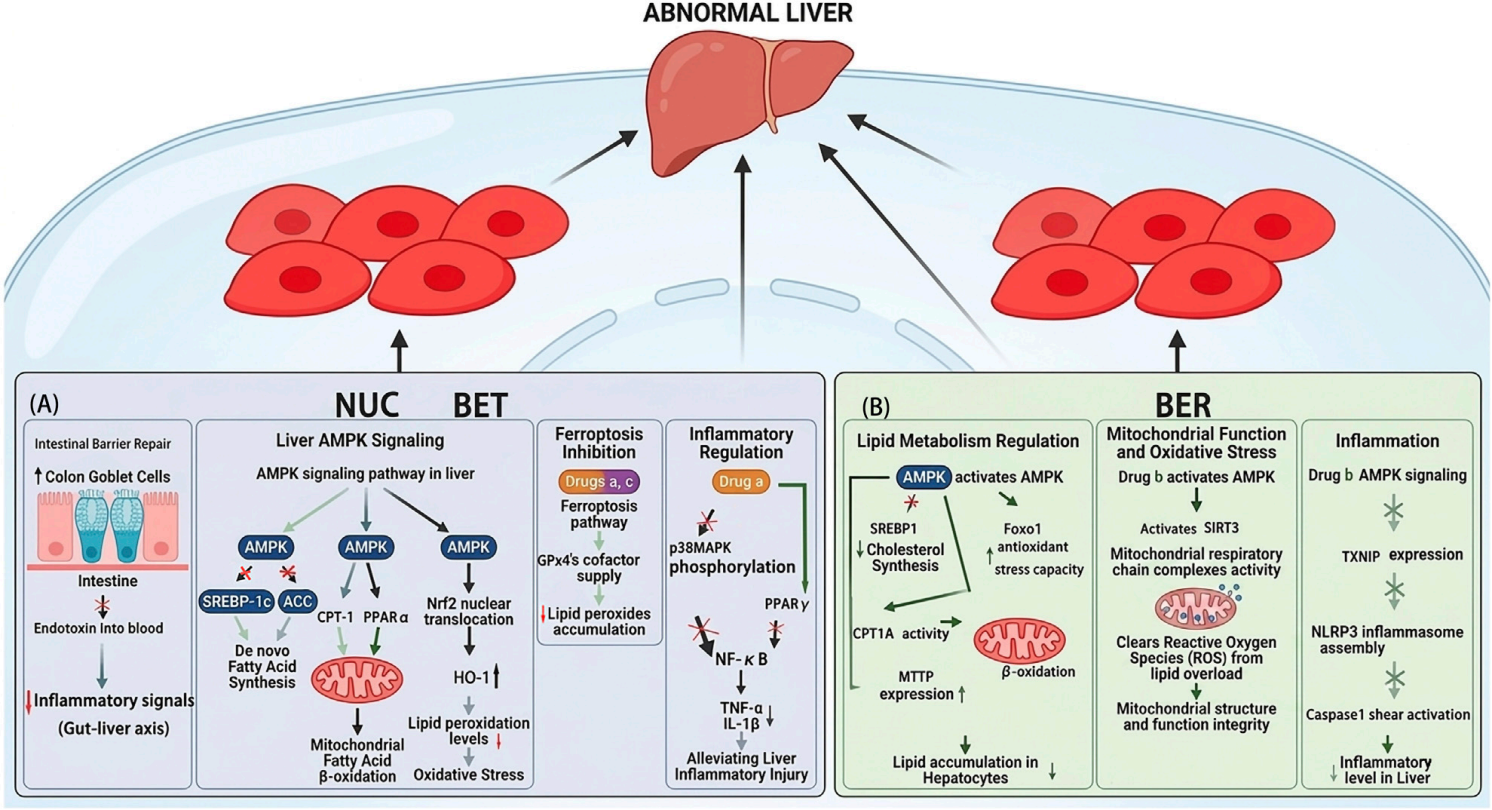
Molecular mechanisms of NUC, BER, and BET in ameliorating hepatic inflammation and lipid metabolism. **(A)** Mechanistic interventions of NUC and BET. NUC and BET abrogate the translocation of endotoxins into the systemic circulation by restoring intestinal barrier integrity (specifically by augmenting goblet cell populations), thereby mitigating inflammation along the gut-liver axis. Mechanistically, these agents activate hepatic AMPK and inhibit the SREBP-1c/ACC pathway to suppress fatty acid synthesis, while upregulating the PPARα/CPT1 axis to promote β-oxidation; this subsequently activates the Nrf2/HO-1 pathway to alleviate oxidative stress. Furthermore, they enhance GPx4 cofactors to indirectly prevent ferroptosis. Simultaneously, they inhibit p38 MAPK phosphorylation and upregulate PPARγ, employing a dual-pathway mechanism to suppress NF-κB activation and attenuate pro-inflammatory cytokine levels. **(B)** AMPK-mediated mechanisms of BER. BER exerts its pharmacological effects primarily via the AMPK pathway. It suppresses SREBP-1, activates FoxO1, enhances CPT1A, and upregulates MTTP, effectively attenuating lipid accumulation in hepatocytes. Moreover, the BER-mediated activation of SIRT3 enhances fatty acid oxidation, thereby maintaining mitochondrial function and energy homeostasis. Finally, BER inhibits TXNIP, which blocks the assembly of the NLRP3 inflammasome and the subsequent activation of caspase-1, thereby comprehensively ameliorating hepatic inflammation.activation of caspase-1, thereby comprehensively ameliorating hepatic inflammation.

Plants synthesize a wide array of nitrogen-based compounds, known as alkaloids, which feature highly varied chemical structures. These compounds, characterized by their intricate polycyclic architectures and abundant functional moieties, including nitrogen-containing heterocycles, hydroxyl groups, and methoxy groups, exhibit great potential for multi-target modulation (Bowman et al., 2025; Grigoriev et al., 2025). Given these characteristics, numerous alkaloid compounds with distinct structural features have demonstrated great potential for treating MASLD/MASH. Preclinical investigations using cellular, zebrafish, and rodent models have revealed that certain alkaloids exhibit multi-target therapeutic effects, including reducing liver lipid deposition, inhibiting oxidative stress, and relieving inflammatory responses. Specifically, these bioactive substances bring about their therapeutic benefits by regulating several key signaling cascades, including PI3K/AKT, AMPK, NF-κB, the NLRP3 inflammasome, mitochondrial autophagy, and the gut microbiota-metabolism axis. This coordinated regulation subsequently contributes to enhancing liver lipid metabolism, IR, and halting the advancement of fibrosis (Figure 2). Among these, AMPK acts as a core energy sensor, playing a pivotal role in the amelioration of lipid metabolism disorders, induction of autophagy, and inhibition of inflammation. Furthermore, certain alkaloids can inhibit hepatocyte ferroptosis by directly scavenging ROS, restricting lipid peroxidation substrate production, and upregulating antioxidant defense factors like PPARα and GPX4. However, the above effects have not been consistently confirmed in all the alkaloids discussed. The differences in specific mechanisms still require further comparative studies. Besides their liver-protective effects, these compounds have also attracted widespread attention due to their anti-tumor, anti-inflammatory, antibacterial, and neurocardiovascular regulatory activities, and they are generally derived from plants with good safety profiles (Kumar et al., 2023). In conclusion, alkaloid-type natural products represent a highly promising class of candidate drug molecules for MASLD, as they intervene in its pathological progression through multiple pathways and targets (Table 1).

**TABLE 1:** Various structural types of alkaloids and their mechanisms of action targeting MASLD.

Structural category	Alkaloids	Source	Structure	Concentration		Duration of study	
				*In vivo*	*In vitro*	*In vivo*	*In vitro*
Organic amine compounds	Betaine (BET)	Natural food	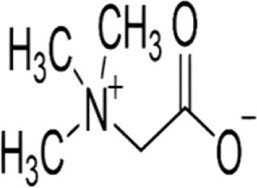	8 g/kg	—	1 week	—
	Capsaicin (CAP)	Capsicum plants	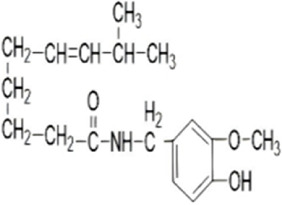	5 mg/kg	50 μM	9 weeks	12–18 h
	Kukoamine A (KuA)	Goji berry (Lycium chinense Miller)	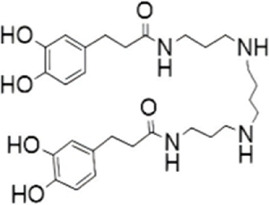	10 mg/kg	—	16 weeks	24 h
	Leonurine (LEO)	labiate	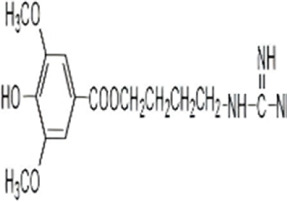	30 mg/kg/d	10 µM	12 weeks	12–14 h
Piperidines	1- Deoxynosphycin (DNJ)	Leaves of the mulberry tree (Morus alba L.)	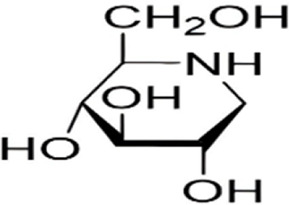	100 mg/kg/d	300 μM	6 weeks	24 h
	Nicotine (NIC)	tobacco	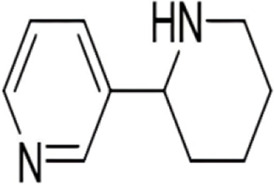	6 mg/kg/d	—	18 weeks	—
	Piperine (PIP)	Pepper plants	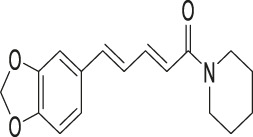	60 mg/kg	50 μg/mL	8 days	24 h
	Sophocarpine (SOP)	foxtail-like sophora herb and seed	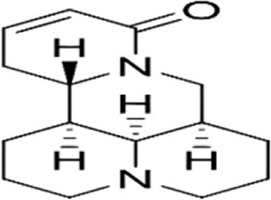	20 mg/kg	200 μmol/L	12 weeks	24 h
	Sangzhiine (SZ-A)	Santalaceae plants	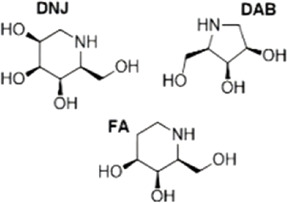	200 mg/kg/d	25 μg/mL	6 weeks	24 h
Isoquinoline compounds	Berberine (BER)	Coptis chinensis	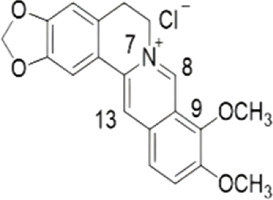	100 mg/kg/d	1 μM	12 weeks	24 h
	Demethyleneberberine (DMB)	Coptis chinensis	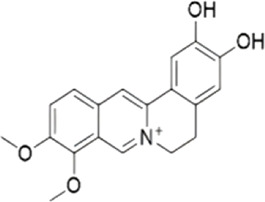	25–100 mg/kg	5–40 μM	6–24 h	6–24 h
	De-methylated tetrahydroberberine (DMTHB)	Derivatives of BBR	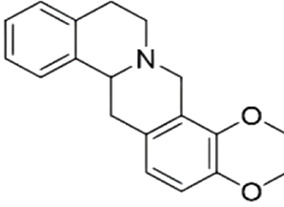	50 mg/kg 150 mg/kg	—	4 weeks	—
	Nuciferine (NUC)	lotus leaf	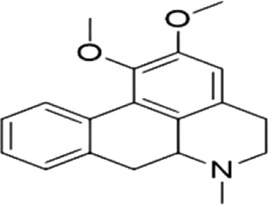	—	10–40 μM	4 weeks	—
	Neferine (NEF)	Nelumbinis plumula	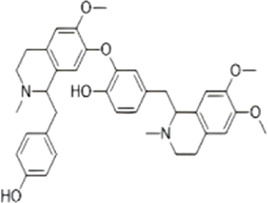	5 mg/kg 10 mg/kg	12.5 μM	4 weeks	24 h
Xanthine compounds	Pentoxifylline (PTX)	methylxanthine derivative	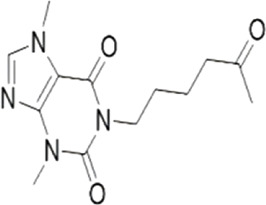	—	2.0 mM	—	16–24 h
	Caffeine (CAF)	methylxanthine	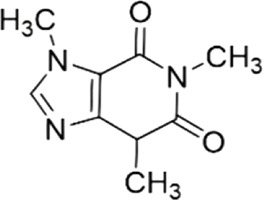	75 mg/kg/d	—	6 weeks	—
Quinolizidine	Oxymatrine (Mat)	Sophora flavescens	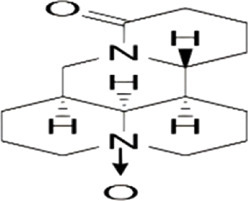	80 mg/kg/d	0.2 mg/mL	4 weeks	24 h
Pyridine	Arecoline (Are)	betel nut	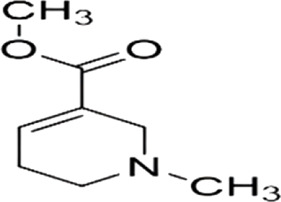	1.5 mg/kg	—	9 weeks	—
	Trigonelline (Tri)	fenugreek	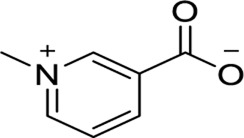	—	75 μM	—	1 week
Indoles	Evodiamine (EVO)	Evodia rutaecarpa Benth	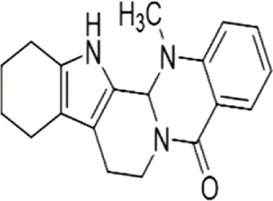	800 mg/kg/d 1,600 mg/kg/d	0.04–25 μmol/L	1 week	48 h
	Hirsutine (Hir)	Uncaria rhynchophylla	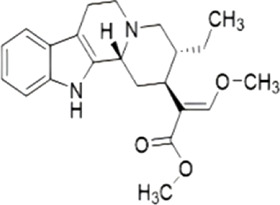	10 mg/kg/d	1 μM	8 weeks	24 h
Pyrrolidines	Stachydrine (Sta)	Rutaceae plants and other related plants	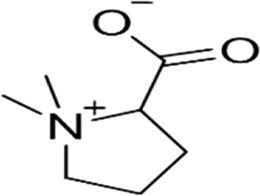	5 mg/kg/d 10 mg/kg/d	20 μM	6 weeks	48 h
Diterpene alkaloids	Brunodelphinine A (BruA)	Delphinium brunonianum Royle	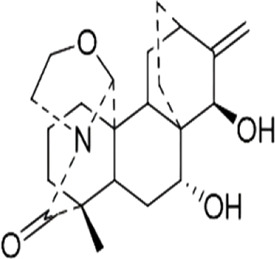	10 mg/kg/d	10 μM	6 weeks	24 h
diketopiperazine dimer	Eurocristatine (ECT)	Eurotium cristatum	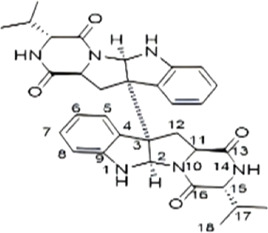	40 mg/kg	30 μM	6 weeks	24 h

Model		Pharmacologic action	Target	Key signaling pathways	Toxicological characteristics	Level of evidence	Conversion status/application	References
*In vivo*	*In vitro*							
Hy-Line Brown laying hens	—	Metabolic diseases, Anti-inflammation	NMDA receptor, TBK1 kinase	JNK/STAT3 pathway	Lower; mild and transient gastrointestinal symptoms	basic research	Preclinical research phase	Wang et al. (2024)
HFFD-induced mice	OA-induced HepG2 cells	Reduce the content of triglycerides	CPT1, PPARδ	Liver transient potential capsaicin receptor 1 cation channel (TRPV1)	No obvious toxic manifestations	basic research	Preclinical research phase	Cao T. et al. (2025)
HFD-induced mice	PA-induced AML-12 cells	Improve fatty degeneration, Oxidative stress damage	SREBP1c, ACC, FAS	JAK/STAT3,PI3K/Akt pathway	No obvious toxic manifestations	basic research	Preclinical research phase	Li et al. (2017)
HFHSD-induced mice	APAP-induced MPHs cells	Metabolic diseases, Anti-inflammatory, Antioxidant stress	ADRA1a	AMPK/SCD1 signal axis	No obvious toxic manifestations	basic research	Preclinical research phase	Yu et al. (2023), Fan et al. (2024)
CDAHFD-induced mice	PA-induced HepG2 cells	Alleviate fatty degeneration, Improve insulin resistance	CPT1,Akt/GSK-3β	PI3K/AKT/mTOR pathway	No obvious toxic manifestations	basic research	Preclinical research phase	Wang Y. Y. et al. (2025), Yang et al. (2026)
PND2-induced Wistar rats	—	Metabolic diseases, Anti-inflammation	PKB	AMPK,PPARα signal pathway	Sex-dependent (males are more susceptible)	basic research	Preclinical research phase	Bertasso et al. (2020), Rivera et al. (2024)
HFD-induced Wistar rats	HepG2 cells	Metabolic diseases, Anti-inflammation	PPARγ,SREBP-1c	AMPK,PPARδ	No obvious toxic manifestations	basic research	Early transformation stage	(Dey et al., 2020; Li S. et al., 2021
SD rats	OA-induced primary hepatocytes	Metabolic diseases, Anti-inflammatory	TLR4, SREBP-1c	NF-κB inflammatory pathway	No obvious toxic manifestations	basic research	Preclinical research phase	Song and Zeng (2021)
HFD-induced mice	PA-induced HepG2 cells	Alleviate fatty degeneration	CD36,PPARγ	AMPK,CD36/PPARγ pathway	No obvious toxic manifestations	basic research	Natural source hypoglycemic drugs	Sun et al. (2022), Liu et al. (2023)
HFD-induced mice	PA/OA-induced HepG2 cells	Cell metabolism, Inflammation	Nrf2, FXR	AMPK,P2X7/NLRP3 inflammatory pathway	No obvious toxic manifestations	basic research	Preclinical research phase	Li et al. (2025)
Con A-induced mice	Con A-induced HepG2/L02 cells	Metabolic diseases, Anti-fibrotic damage	MDA,GSH	AMPK,NF-κB,TGFβ-Smad pathway	No obvious toxic manifestations	basic research	Preclinical research phase	Zhang et al. (2020c)
MCD-induced mice	PA-induced L02 cells	Metabolic diseases, Anti-fibrotic damage	NLRP3,TLR4	NLRP3/CYP2E1/ATF-4 pathway	No obvious toxic manifestations	basic research	Preclinical research phase	Zhang et al. (2021)
MCD-induced mice	FFA-induced HepG2 cells	Lipid metabolism, Inflammation	FAT,SREBP-1c	PPARα/γ,TLR4/NF-κB/NLRP3 signal	No obvious toxic manifestations	basic research	Preclinical research phase	Zhao et al. (2025)
HFD + CCl_4_-induced mice	OA-induced HepG2 cells	Improve liver steatosis, liver fibrosis	AMPK,ACC,CPT1A,SREBP1	AMPK signal pathway	No obvious toxic manifestations	basic research	Preclinical research phase	Wang et al. (2023)
—	Mouse oocyte IVM	Liver steatosis, Fibrosis damage	GRP78,ATF4,ATF6	NF-κB,MAPK signal pathway	No obvious toxic manifestations	Class I/II clinical studies	Approved drugs	Zein et al. (2012), Wu et al. (2024)
HFHC-induced mice	—	Promote fat breakdown, Inhibit synthesis	PPARγ,Nrf2	AMPK,PI3K/Akt,Nrf2-AREsignal pathway	No obvious toxic manifestations	basic research	Preclinical research phase	Xin et al. (2021)
HFDHFr-induced Wistar rats	PA-induced HepG2 cells	Improving insulin resistance, Inhibiting fat synthesis	CPT1A,SREBP-1,ACC	SIRT1/AMPK,PPARα signal pathway	No obvious toxic manifestations	basic research	Preclinical research phase	Zhang et al. (2020b)
Ctenopharyngodon idella	—	Gut microbiota, Lipid metabolism	COX2,PGE2	ABCA1/LXRα,FoxO1/PGC-1α signal pathway	dose-dependent relationship	basic research	Preclinical research phase	Cao Z. J. et al. (2025), Wu et al. (2025)
—	3T3-L1 cells	Improving insulin resistance, Inhibiting fat synthesis	PPARγ, C/EBPα	SREBP-1c,AMPK/SIRT1pathway	No obvious toxic manifestations	basic research	Preclinical research phase	Choi et al. (2020)
Normal mice	HepG2 cells	Regulate the intestinal flora, Inflammation	ABCA1,TRPV1	TLR4/NF-κB pathway	dose-dependent relationship	basic research	Preclinical research phase	Tao et al. (2025), Zhang et al. (2025)
HFD-induced mice	HGHI-induced HepG2 cells	Improve insulin resistance in the liver and heart	p-Akt,GLUT4	PI3K/Akt,AMPK pathway	No obvious toxic manifestations	basic research	Preclinical research phase	Hu et al. (2022)
HFD-induced mice	3T3-L1 cells	Cell autophagy, Oxidative stress	Srebp-1c, PPARγ	AMPK/SIRT1 signal pathway	No obvious toxic manifestations	basic research	Preclinical research phase	Lee et al. (2022)
HFD-induced mice	FFA-induced HepG2 cells	Metabolic diseases, Reduction of lipid accumulation	NOX4	NOX4/SIRT1/PPARs signal axis	No obvious toxic manifestations	basic research	Preclinical research phase	Wang M. Q. et al. (2025)
db/db mice	High glucose-induced HepG2 cells	Alleviate insulin resistance	PI3K	PI3K/AKT signaling pathway	No obvious toxic manifestations	basic research	Preclinical research phase	Zhang et al. (2020a)

The fourth part of this article will focus on the discussion of three alkaloids: BER, NUC, and BET. The main considerations for this discussion are as follows: Firstly, the three belong to different chemical subcategories of alkaloids. BER and NUC both belong to the isoquinoline class but have different subtypes: BER is the original berberine type, while NUC is the aporphine type; BET is the quaternary ammonium type. This structural diversity is conducive to systematically comparing the structure-activity relationships of the alkaloid family. Secondly, the three have different focuses and complementary mechanisms in their MASLD-related pharmacological effects. BER mainly improves metabolic disorders by activating the AMPK pathway and regulating the intestinal microbiota; NUC shows broad activity in anti-inflammation, anti-atherosclerosis, improvement of fatty liver, and cancer prevention; while BET participates in one-carbon metabolism and epigenetic regulation as a methyl donor. The differentiated mechanisms of the three precisely constitute a complete comparison framework from energy metabolism and inflammation regulation to methylation modification. Furthermore, the three represent different research stages. BER has entered the clinical trial stage (Mohapatra et al., 2026), while NUC and BET are currently mainly in the preclinical research stage. This difference enables the comparison of the three to not only summarize the clinical experience achieved but also provide directions for subsequent new drug development. In conclusion, the triple representativeness makes the comparison of these three alkaloids have both structural taxonomic significance and pharmacological mechanism value, and can also provide a step-by-step reference framework for the clinical transformation of natural products.

### 4.1 Berberine

Berberine (BER), an isoquinoline alkaloid, is derived from the stems and roots of various berberis plants. It is also known to have a favorable safety profile and has garnered significant attention as a key focus in natural products research (Ming et al., 2023). Additionally, BER, existing as a bitter, crystalline yellow powder in its free form, shows poor solubility in water and low-polarity organic solvents, though it dissolves well in hot ethanol (Feng et al., 2019). With a molecular formula of C_20_H_18_NO_4_, its chemical structure comprises an isoquinoline ring skeleton, methoxy groups (-OCH_3_), a methylenedioxy group (-O-CH_2_-O-), and a quaternary ammonium group (N^+^) (Wang L. et al., 2023). Notably, the methylenedioxy group enhances molecular rigidity, which facilitates its intercalation into hydrophobic regions to exert anti-inflammatory activities (Nguyen et al., 2023). BER, a versatile compound long utilized in traditional herbal medicine, exhibits a broad spectrum of biological activities, such as anti-cancer, anti-inflammatory, cardioprotective, and free radical-scavenging effects. Beyond its well-documented roles in cancer and inflammation, BER also exhibits significant potential for liver health by interacting with multiple biological pathways, thereby showing promise in treating diverse liver disorders and maintaining metabolic balance (Yang et al., 2022). Consequently, it has emerged as one of the most thoroughly studied substances within the domains of metabolic and liver-related conditions.

In animal model studies of the gut-liver axis and the intestinal microbiota, the oral administration of BER primarily reinforces the gut barrier and reshapes microecological homeostasis through a multi-target synergistic mechanism, rather than relying on high systemic exposure. Initially, BER upregulates Tj proteins (occludin, claudin, ZO-1) to enhance the integrity of the intestinal epithelial barrier. Concurrently, by modulating the “gut microbiota - short-chain fatty acids (SCFAs) - LPS - IL-6 - signal transducer and activator of transcription 3 (STAT3)” axis, it increases the abundance of SCFA-producing bacteria, reduces LPS levels, and inhibits STAT3 and IL-6, thereby alleviating inflammation and blocking bacterial translocation (Wang et al., 2026). Furthermore, BER inhibits microbial bile salt hydrolase (BSH) activity, leading to the accumulation of conjugated bile acids (such as taurocholic acid, TCA). This accumulation activates FXR and its downstream Fibroblast Growth Factor 15 (FGF15) signaling and subsequently exerts feedback inhibition on liver CD36 expression via the intestine-liver axis, ultimately improving lipid metabolism (Tian et al., 2026). Meanwhile, this process stimulates the proliferation of *Acetobacter* while suppressing AMPK, leading to an increase in cholesterol 7α-hydroxylase (CYP7A1) expression, which in turn facilitates the conversion of cholesterol into bile acids (Hang et al., 2025). Integrated multi-omics analysis confirms that the BSH-TCA-FXR-FGF15 cascade reaction is highly consistent with transcriptomic changes in the liver, suggesting that the primary therapeutic targets of BER are located within the intestinal microecological niche (Song et al., 2025). This insight further explains the compound’s effectiveness despite its limited bioavailability. Inflammatory regulation by BER involves the suppression of the NF-κB pro-inflammatory pathway, downregulation of cyclooxygenase-2 (COX-2), TNF-α, IL-1β, and IL-6, and activation of the Nrf2/HO-1 antioxidant axis (Aghili et al., 2024). Notably, in the liver injury model, there is an interactive regulation of NF-κB and Nrf2 by BER, jointly constituting a regulatory network that balances inflammation and oxidative stress.

Additionally, BER modulates the execution of diverse cell death patterns, as exemplified by its role in D-GalN/LPS-induced acute liver failure where pretreatment alleviates hepatocyte apoptosis through suppressing TNF-α production, halting mitochondrial cytochrome c release, lowering the Bax/Bcl-2 expression ratio, and inhibiting caspase-9/3 activation (Xu et al., 2018). Furthermore, BER upregulates deacetylase sirtuin 1 (SIRT1) to resist hydrogen peroxide-induced apoptosis and regulates circular RNA circDNTTIP2 to inhibit its binding to the caspase-3 promoter, thereby reducing caspase-3-dependent apoptosis (Zhu Y. et al., 2023). Additionally, BER may also exert indirect regulatory effects on ferroptosis and pyroptosis, although direct evidence remains to be established. By leveraging this positive charge, the quaternary ammonium compound not only directs BER to accumulate within mitochondria to activate AMPK, but also enables the selective delivery of antioxidants or ferroptosis inhibitors to mitochondria, thereby offering precise control over lipid peroxidation and iron buildup. This targeted strategy not only prevents MASLD from progressing to MASH and fibrosis but also minimizes systemic side effects and can be tailored to individual metabolic conditions for personalized treatment (Luo et al., 2024).

BER promotes the phosphorylation of AMPK Thr^172^ through a multi-tiered network. Notably, LKB1 is the most definitively identified upstream target: BER enhances LKB1 phosphorylation, which in turn phosphorylates the Thr^172^ residue of the AMPKα subunit site to initiate AMPK activation (Zhu et al., 2025; Guo X. Y. et al., 2026). Upstream metabolic signals are also involved in this process. For instance, in high-fructose-induced IR mice, BER activates the liver LKB1/AMPK/PGC1α pathway to improve mitochondrial function; conversely, BER in the Huanglian Wendan Decoction activates LKB1/AMPK by increasing *Akkermansia* abundance and modulating aspartic acid metabolism, thereby initiating carnitine palmitoyltransferase 1A (CPT1A)- and PPARα-mediated fatty acid oxidation to alleviate lipid degeneration. In addition, BER reduces triglyceride synthesis by activating the AMPK-SREBP-1c-stearoyl-CoA desaturase 1 (SCD1) pathway (Jiang et al., 2025). Upon activation, AMPK not only inhibits SREBP-1c and SCD1 to reduce *de novo* lipid synthesis but also upregulates CPT1A/PPARα to promote β-oxidation. These dual effects have been validated in HepG2 cells, 3T3-L1 cells, and various animal models, effectively mitigating lipid degeneration. The activated AMPK further enhances antioxidant capacity, upregulates microsomal triglyceride transfer protein (MTTP) by activating forkhead box protein O1 (FoxO1), and alleviates intracellular lipid deposition in liver cells by enhancing very low-density lipoprotein (VLDL)-mediated lipid efflux. Concurrently, it activates deacetylase 3 (SIRT3) to elevate respiratory chain activity and fatty acid oxidation, facilitating the clearance of ROS and maintaining mitochondrial homeostasis (Hu et al., 2024). More crucially, AMPK inhibits thioredoxin-interacting protein (TXNIP) expression and blocks the assembly of the NLRP3 inflammasome, activation of caspase-1, and pore formation by GSDMD, leading to a significant reduction in the level of liver inflammation (Chang, 2024).

Although BER can exert anti-inflammatory and anti-fibrotic effects by modulating the NF-κB, LKB1/AMPK/PGC1α, and gut microbiota-SCFA-LPS/IL-6/STAT3 axes *in vitro* and in animal models, some studies have noted that despite being supported by level 1 imaging evidence (Lei et al., 2026), it remains hindered by limitations such as a moderate effect size, high formulation heterogeneity, and a lack of histological confirmation. The oral bioavailability of BER is less than 1%, and the concentrations employed in most mechanistic studies far exceed actual human exposure limits. The animal doses (25–100 mg/kg) are also significantly higher than clinical dosages, making it uncertain whether the target effects are reproducible in humans (Sai Durga Surampudi et al., 2025; Yang X. et al., 2025). In addition, although BER can promote beneficial bacteria such as *Akkermansia*, it simultaneously induces the formation of *Escherichia coli* biofilms, leaving long-term microecological risks to be clarified (Wang Y. F. et al., 2025). Regarding the pharmacological safety of BER, current literature indicates that it harbors risks of genotoxicity, drug interactions, and cytotoxicity, while concurrently possessing renoprotective and neuroprotective properties (Abrams et al., 2019; Rezaee et al., 2019). Consequently, its safety is contingent upon dosage, individual variations, concomitant medications, and disease status, necessitating systematic evaluation. In summary, current evidence presents notable deficiencies regarding the disconnect between *in vivo* drug exposure and efficacy, methodological standardization, risk of resistance, and safe dosing regimens; thus, its indiscriminate promotion lacks sufficient justification. Future endeavors should prioritize conducting multicenter randomized controlled trials, optimizing pharmaceutical formulations to enhance bioavailability, and exploring personalized strategies across different MASLD subtypes.

### 4.2 Nuciferine

Nuciferine (NUC) is an aporphine alkaloid predominantly extracted from the leaves of the lotus plant (*Nelumbo nucifera*). Its molecular formula is C_19_H_21_NO_2_, and its structural scaffold typically features functional groups such as aromatic rings and methoxy groups (-OCH_3_) integrated into an aporphine backbone (Ren et al., 2024). Among these, the aromatic ring is critical for the recognition and activation of multiple key molecular targets, such as AMPK and PPARα, which collectively orchestrate the broad regulation of lipid metabolism (Li J. X. et al., 2023). Modern pharmacological studies indicate that NUC possesses a diverse array of biological activities, including anti-inflammatory, anti-obesity, hypolipidemic, insulin-sensitizing, and anti-tumor effects, with no significant toxicity observed (Qi et al., 2016). Clinical trials have further demonstrated that aqueous lotus leaf extracts significantly ameliorate dyslipidemia in hyperlipidemic patients, evidenced by decreased levels of total cholesterol (TC), TG, and low-density lipoprotein cholesterol (LDL-C), alongside increased levels of high-density lipoprotein cholesterol (HDL-C) (Zhang et al., 2022).

In lab investigations, researchers found that NUC effectively lowers triglyceride levels in 3T3-L1 fat cells and enhances lipid processing, with this beneficial action largely relying on the promotion of AMPK phosphorylation. Specifically, activated AMPK phosphorylates and inhibits ACC, thereby reducing malonyl-CoA synthesis. In this context, AMPK activation also interferes with triglyceride production while alleviating the allosteric suppression of CPT1, thus boosting cellular fatty acid β-oxidation (Zhu X. Y. et al., 2023). In a similar vein, AMPK exerts its metabolic effects by not only stimulating the breakdown of stored fats through hormone-sensitive lipase (HSL) and boosting the expression of genes like *PPARα* but also improving insulin responsiveness, which collectively helps lower fat build-up in fat cells and increase energy usage in various ways. Notably, NUC also robustly activates the hepatic AMPK signaling pathway. On the one hand, activated AMPK suppresses *de novo* lipogenesis by inhibiting SREBP-1c and ACC; on the other hand, it activates PPARα protein and CPT1 to enhance mitochondrial fatty acid β-oxidation. Additionally, AMPK activation promotes Nrf2’s entry into the nucleus, increasing the production of antioxidant enzymes like HO-1. This process not only directly reduces lipid peroxidation but also mitigates oxidative stress (Guo Y. et al., 2026).

Regarding inflammatory cascades, NUC exerts potent anti-inflammatory effects through multi-target regulation. NUC also demonstrates robust anti-inflammatory actions via its ability to regulate multiple pathways, specifically by reducing the phosphorylation and subsequent breakdown of NF-κB inhibitor α (IκBα), which in turn blocks the movement of the NF-κB p65 subunit from the cytoplasm to the nucleus. As a result, this action curbs the transcriptional activity of NF-κB and decreases the expression of pro-inflammatory cytokines including TNF-α, IL-6, and IL-8. Concurrently, NUC significantly upregulates hepatic SOD and total antioxidant capacity (T-AOC), thereby reducing the accumulation of the lipid peroxidation product malondialdehyde (MDA) and effectively mitigating oxidative stress (Zhao et al., 2023). Furthermore, NUC blocks LPS-induced inflammatory responses by inhibiting the p38 MAPK/ATF2 pathway (Kim et al., 2022); it concurrently inhibits p38 MAPK phosphorylation while upregulating PPARγ expression. These dual mechanisms synergistically block NF-κB activation, curtailing the release of key pro-inflammatory mediators such as TNF-α and IL-1β, thereby effectively mitigating hepatic inflammatory damage through three distinct pathways: halting the onset of inflammatory signaling, suppressing transcription factor activation, and boosting intrinsic antioxidant capabilities.

The hepatoprotective efficacy of NUC is intimately linked to its regulation of two distinct cell death modalities. First, NUC directly binds to the Ragulator complex on the lysosomal surface, impeding the lysosomal recruitment and activation of mTORC1; this subsequently disinhibits transcription factor EB (TFEB) and enhances the functionality of the autophagy-lysosome pathway (Du et al., 2022). Second, NUC significantly inhibits ferroptosis in a manner dependent on the activation of the PPARα signaling pathway. Following PPARα protein activation, NUC upregulates CPT1A to facilitate the transport of fatty acids into mitochondria for β-oxidation, thereby fundamentally depleting the free fatty acid substrates required for triglyceride synthesis and lipid peroxidation. It simultaneously upregulates GPX4, an essential enzyme that reduces toxic lipid peroxides into non-toxic lipid alcohols. Consequently, NUC directly fortifies cellular resistance against lipid peroxidation and ferroptosis via the PPARα/GPX4 axis (Qiu et al., 2024).

Moreover, NUC contributes to decreasing lipid buildup in liver cells and enhancing IR through two closely associated mechanisms. One critical mechanism by which NUC exerts its effects is its capacity to repress the expression of the E3 ubiquitin ligase MIB2. This action prevents the K48-linked ubiquitination and subsequent breakdown of the Caspase recruitment domain-containing protein 6 (CARD6) protein. As a result, CARD6 remains stable, which in turn blocks the activation of downstream apoptosis signal-regulating kinase 1 (ASK1), decreases the phosphorylation levels of JNK1/2, and finally limits hepatic lipid synthesis, thus relieving hepatic steatosis (Li F. et al., 2023). Furthermore, NUC can positively regulate the activity of insulin receptor substrate 1 (IRS1) and enhance the transmission of PI3K/Akt signaling pathway. This, in turn, promotes cellular glucose uptake, suppresses hepatic glucose synthesis via gluconeogenesis, and enhances the body’s insulin sensitivity (Le et al., 2021). Crucially, the JNK pathway’s overactivation can impair IRS1 function, so by blocking JNK signaling with NUC, the body’s insulin sensitivity is indirectly strengthened. Additionally, the reduction in intrahepatic lipid accumulation further optimizes the microenvironment for insulin signaling.

To summarize, NUC demonstrates a range of pharmacological actions targeting multiple pathways, such as anti-inflammatory, vascular-protective, anti-atherosclerotic, and metabolic-regulatory effects (Li D. Y. et al., 2021). However, the existing data mainly come from *in vitro* and rodent experiments, and high-quality clinical evidence remains insufficient. This limitation presents three key translational challenges. To begin with, NUC exhibits very low oral absorption, with an absolute bioavailability of just 1.9% (Ding et al., 2025), yet it still produces notable therapeutic effects even at low concentrations, creating a discrepancy between its pharmacokinetic properties and pharmacological outcomes. This apparent contradiction implies that its *in vivo* efficacy could be regulated by gut microorganisms or active metabolites (Yu et al., 2021). Second, species-specific variations in metabolic processes limit the clinical relevance of preclinical findings. Third, despite mechanistic investigations pointing to several pathways, including TLR4/NF-κB and PPARα/PGC1α, the fundamental underlying mechanism remains elusive because of substantial differences across experimental models and dosages. Furthermore, the potential toxicity and adverse effects of NUC primarily include time- and dose-dependent cytotoxicity in certain cell lines (e.g., HepG2) (Li T. T. et al., 2025), as well as strong antagonistic effects on β_2_-adrenergic, 5-Hydroxytryptamine Receptor 2 (5-HT_2_), and α_1_ receptors. These opposing effects may also trigger unwanted responses, such as difficulty breathing, confusion, low blood pressure when standing, and lightheadedness (Zhang et al., 2018). In conclusion, while NUC shows potential as a natural compound, it still encounters major challenges in moving from lab to clinic. Moving forward, addressing the pharmacokinetic-pharmacodynamic contradiction and confirming its primary mechanisms in human-oriented models will be critical areas for investigation.

### 4.3 Betaine

Betaine (BET), also known as trimethylglycine, is a stable, non-toxic, naturally occurring zwitterionic compound. Its molecular formula is C_5_H_11_NO_2_, and its structural scaffold features two primary functional groups: a carboxyl group (-COOH) and a quaternary ammonium group (-N(CH_3_)^3+^). Widely distributed across both the animal and plant kingdoms, BET can be synthesized endogenously through choline metabolism or acquired exogenously via dietary intake (Willingham et al., 2020; Zhang Y.-Y. et al., 2025). Within food matrices, its highest concentrations are found in whole grains (e.g., whole wheat flour, bread, and pasta), select vegetables (e.g., spinach and beetroot), and various marine shellfish (e.g., clams and scallops) (Dobrijević et al., 2023). As a highly water-soluble compound, BET serves alongside folate derivatives as one of the primary metabolic sources capable of providing methyl groups for the remethylation of homocysteine. Additionally, it functions as a critical osmoprotectant, assisting cells in maintaining osmotic balance and resisting environmental stressors such as thermal and hyperosmotic shock (Essawy et al., 2025). Compared to its synthetic analogs, natural BET is heavily favored in pharmaceutical, cosmetic, and nutraceutical applications due to its superior functional profile. BET exhibits pleiotropic pharmacological properties (Altinisik et al., 2023), including anti-inflammatory, antioxidant, anti-epileptic, neuroprotective, and anti-depressant effects, while also demonstrating clinical utility in managing obesity, hepatic disorders, cardiovascular diseases, and specific malignancies (Du et al., 2021). *In vivo* studies have confirmed its efficacy in optimizing body composition, specifically by reducing adiposity and promoting muscle accretion, through the intricate regulation of fatty acid metabolism (i.e., enhancing lipid oxidation while suppressing lipogenesis) (Kuerbanjiang et al., 2024).

Mechanistic investigations have revealed that BET, while functioning as a methyl donor to restore the liver’s methylation potential (Chen et al., 2022), does not directly activate AMPK but instead operates through an indirect pathway involving fibroblast growth factor 10 (FGF10) secreted by liver cells (Chen et al., 2021): BET promotes the secretion of FGF10 from liver cells. Once released, FGF10 interacts with its specific receptors FGFR1 and FGFR2 on the surface of liver cells, triggering a series of intracellular signaling events that culminate in the activation of LKB1, a key upstream kinase in the AMPK pathway. This indirectly triggers LKB1-mediated phosphorylation of AMPK at Thr^172^, thereby activating AMPK without direct binding. Once active, AMPK exhibits two key functions: on the one hand, it suppresses the activity of SREBP-1c and its downstream target genes like FASN and ACC, thereby preventing the synthesis of new lipids. On the other hand, it enhances the expression of Adipose triglyceride lipase (ATGL) to facilitate lipid breakdown. Concurrently, BET restores the expression or activity of PPARα, further enhancing fatty acid β-oxidation and promoting the secretion of VLDL, thus alleviating lipid accumulation in the liver through multiple pathways (Rehman and Mehta, 2022). Complementary studies have validated that elevating FGF10 expression independently replicates BET’s observed benefits, decreasing liver lipid deposits and boosting AMPK activation. Conversely, impairing this signaling cascade diminishes its beneficial impact on lipid metabolism. Thus, BET exerts a synergistic regulatory role on lipid metabolism, encompassing synthesis, degradation, and transport, through the FGF10-FGFR1/2-LKB1-AMPK signaling pathway, ultimately alleviating MASLD-associated metabolic dysfunction.

In the context of inflammatory responses triggered by alcohol exposure or tissue damage, BET proteins exert their anti-inflammatory effects by inhibiting the expression of several key inflammatory factors, with nitric oxide synthase 2 (NOS2) being a primary target. By curbing the overproduction of nitric oxide (NO) and peroxynitrite, BET helps mitigate oxidative and nitrosative stress to mitochondria, thereby maintaining the energy balance of hepatocytes (Arumugam et al., 2021). Upon hepatocellular injury, the released high-mobility group box 1 (HMGB1) protein binds to TLR4 receptors expressed on the surface of Kupffer cells, which subsequently activates the NF-κB pathway via a MyD88-dependent signaling cascade. This activation drives the transcription of key pro-inflammatory cytokines such as TNF-α, IL-1β, IL-6, and NOS2. In this context, BET acts as a potent disruptor of the HMGB1–TLR4 interaction and effectively inhibits the downstream MyD88/NF-κB signaling axis, thereby systematically quelling the hepatic inflammatory response at its earliest source. The persistence of such an inflammatory microenvironment often triggers the activation of HSCs, promoting their transdifferentiation into myofibroblasts that highly express extracellular matrix proteins, most notably α-Smooth Muscle Actin (α-SMA) and type I collagen, ultimately culminating in hepatic fibrosis. Remarkably, BET modulates this fibrotic process via a two-pronged approach: by alleviating hepatic inflammation as a secondary effect, and likely by directly influencing HSCs to repress the production of α-SMA, type I collagen, and TGF-β. BET, in this manner, not only curbs extracellular matrix buildup but also retards the progression of fibrogenesis. Taken together, BET establishes a comprehensive, multi-target hepatoprotective network that seamlessly integrates mitochondrial protection, inflammatory blockade, and robust anti-fibrotic activity.

Emerging research indicates that incorporating 1% BET into the diet of pregnant and lactating women consuming a high-fat diet (HFD) can significantly alter the gut microbiota structure of their infants. This BET intervention not only significantly modifies the gut microbial community structure of the offspring but also decreases the proportion of harmful bacteria like *Deinococcus* class and *Desulfurococcus* and *Ruminococcus* genera, while promoting the growth of beneficial groups such as *Bacteroides* and *Prevotella*. Alongside this microbial remodeling, a substantial increase in total fecal SCFAs is observed, with notable elevations in acetate, butyrate, and valerate. Importantly, these shifts in gut microbiota and SCFAs are inversely associated with hepatic triglyceride accumulation in the offspring (Sun et al., 2023). Additionally, in a separate investigation utilizing HFD-fed MASLD mice, a metabolic cofactor combination incorporating BET was found to restore gut microbial balance and boost SCFA concentrations. These adjustments in the gut microbiome and SCFA levels subsequently result in increased expression of *PPARα* and *CPT1A* in the liver, while reducing the expression of the pro-inflammatory cytokine TNF-α. Consequently, these molecular events synergistically enhance lipolysis, dampen inflammatory responses, and ameliorate hepatic steatosis (Quesada-Vázquez et al., 2022). In addition, BET reinforces the integrity of the intestinal physical barrier by upregulating Tj proteins (e.g., ZO-1 and occludin), increasing goblet cell numbers, promoting intestinal epithelial cell proliferation, and reducing intestinal permeability. In summary, such mechanisms serve to block the movement of bacterial substances into the portal blood stream. Meanwhile, BET suppresses the TLR4/MyD88 signaling pathway to preserve microbial homeostasis, thereby acting on multiple nodes of the gut-liver axis to hinder disease progression (Chen et al., 2020).

Rasineni et al. reported that in an LPS/D-GalN-induced acute liver injury model, asialoglycoprotein receptor (ASGP-R)-deficient mice exhibited elevated hepatic caspase-3 activity—indicative of rampant apoptosis—alongside surging serum TNF-α and IL-6 levels, culminating in exacerbated liver damage. Pre-administration of BET significantly attenuated caspase-3 activity and suppressed TNF-α and IL-6 secretion, thereby mitigating hepatocellular apoptosis and inflammatory injury (Rasineni et al., 2020). Regarding ferroptosis, there is currently no direct evidence to suggest that BET exerts an inhibitory effect. Given that BET can activate Nrf2, a key transcription factor that regulates the ferroptosis defense system (e.g., ferritin and GPX4), it is hypothesized that BET may indirectly regulate ferroptosis through this transcriptional network. However, this hypothesis requires validation through direct experimental evidence in future studies (Yang Z.-J. et al., 2025).

The role of BET in lipid metabolism is complex. Numerous animal and cellular studies have indicated that it ameliorates hepatic lipid accumulation and IR by inhibiting fatty acid synthesis, promoting β-oxidation, and regulating methylation. However, contradictory evidence also exists; in avian species, C2C12 cells, and finishing pigs, BET has been shown to promote lipid synthesis and deposition (Liu J. Y. et al., 2022), suggesting that its effects are dependent on tissue, species, and physiological context. Methodological limitations in current research include species heterogeneity, varied experimental durations, and the neglect of gut-liver axis regulation in both *in vivo* and *in vitro* studies. The core controversy lies in the dual lipogenic and anti-lipogenic effects of BET, and its impact on circulating lipid profiles remains inconsistent. Some studies have reported decreases in TC, TG, and LDL, whereas one review suggests that LDL levels might actually be elevated (Blachier et al., 2020). Furthermore, the exploration of BET’s epigenetic mechanisms requires more substantial evidence, and its long-term safety profile needs further clarification. At the translational level, only one 8-week randomized controlled trial (RCT) involving 102 overweight or obese individuals is currently available. Moreover, the genetic background of the subjects, such as *PEMT* and *MTHFR* polymorphisms, may significantly influence therapeutic efficacy (Młodzik-Czyżewska et al., 2021; Zhang et al., 2026), a challenge further compounded by the complex pathology of MASLD. In summary, BET acts as a double-edged sword in lipid regulation. Future research should focus on conducting standardized clinical trials, elucidating tissue-specific differences, and determining a safe dosage window.

### 4.4 Other alkaloids

Beyond the aforementioned compounds, several other alkaloids exhibit multi-target regulatory properties, expanding the therapeutic armamentarium against MASLD. Notable examples include oxymatrine, caffeine, and evodiamine. The therapeutic efficacy of Oxymatrine against MASLD-associated hepatitis relies primarily on synergistic anti-inflammatory and antioxidant cascades. Regarding inflammation, it suppresses the pro-inflammatory cytokine TNF-α while elevating anti-inflammatory factors (IL-10 and IL-27) by upregulating the Stimulator of Interferon Genes (STING)/TANK-Binding Kinase 1 (TBK1)/Interferon Regulatory Factor 3 (IRF3) pathway (Wang S. et al., 2025). Oxidatively, it mitigates MDA and ROS accumulation and augments the activity of antioxidant enzymes, such as SOD and catalase (CAT), via the activation of the Nrf2/HO-1 axis (Kang et al., 2022). Furthermore, a large-scale cohort study encompassing over 180,000 participants from the UK Biobank revealed that individuals consuming more than 2.5 servings of caffeinated coffee daily exhibit a 31% lower risk of developing MASLD (Liu W. Q. et al., 2026). In animal models, caffeine significantly attenuates hepatic steatosis, inflammation, and early-stage fibrosis. A key factor underlying these beneficial outcomes is the increased production of dual-specificity phosphatase 9 (DUSP9) in the liver. When this enzyme is more active, it successfully suppresses the subsequent ASK1-p38/JNK signaling pathway, which helps to reverse issues with both glucose and lipid metabolism while also reducing both inflammatory responses and cell death processes (Xin et al., 2025). In addition, caffeine can also reduce the accumulation of lipids in the liver through its involvement in the TNF and p38 MAPK signaling pathways (Dong et al., 2025). In the context of MASLD, evodiamine exerts its therapeutic effects through a multitargeted regulatory mechanism. When used in combination with BER, it synergistically regulates the gut microbiota, increasing the abundance of beneficial bacteria such as *Lactobacillus* while reducing pathogenic bacteria like *Fusobacterium*. Furthermore, network pharmacology research has identified multiple signaling pathways involved in its action, including those related to protein kinase Bα (AKT1), TNF, insulin, and adipocytokines, which collectively influence critical metabolic processes. Specifically, it regulates fatty acid β-oxidation by targeting acyl-CoA synthetase long-chain family member 1 (ACSL1) and carnitine palmitoyltransferase 1B (CPT1B). Additionally, it modulates cholesterol synthesis, inhibits IL-17/NF-κB inflammatory signaling, and protects the intestinal barrier, thereby ameliorating hepatic steatosis and hepatocellular injury (Gao et al., 2022). These diverse yet coordinated therapeutic actions not only demonstrate the intricate, multi-targeted regulatory properties of plant-derived compounds but also provide essential insights at the cellular level for developing tailored therapies targeting distinct lipid metabolic subtypes. As a result, natural alkaloids present significant synergistic and complementary benefits to the current pharmacological system for managing MASLD.

### 4.5 Structure-activity relationship: structural features and multi-target regulation

While systematic, direct comparative studies on the structure-activity relationship (SAR) of alkaloids in treating MASLD remain limited, analyzing the mechanisms of action of alkaloids with diverse chemical structures elucidates the associations between key structural features and their beneficial activities. Isoquinoline compounds (such as BER) and aporphine compounds (such as NUC) typically possess polycyclic aromatic systems. Among these, BER, an isoquinoline quaternary alkaloid, features a positive charge that enables it to bind to negatively charged molecules (such as DNA or specific carboxyl groups) *in vivo* through ionic interactions. This ionic pairing not only influences its water solubility and stability but may also enhance its bioavailability. Furthermore, the quaternary ammonium cation allows BER to intercalate into DNA, thereby interfering with DNA replication and transcription (Alipour Noghabi et al., 2023). The aromatic aporphine skeleton serves as the core structural basis for the therapeutic efficacy of NUC in MASLD. This skeleton confers gut-targeting properties: on the one hand, it binds to the bitter taste receptor TAS2R46 to stimulate GLP-1 secretion, thereby regulating hepatic VLDL metabolism; on the other hand, its low oral bioavailability results in high localized intestinal concentrations, which paradoxically potentiates its modulatory effects on the gut-liver axis (Ding et al., 2025). Additionally, NUC can directly bind to LKB1 to activate the AMPK/mTOR pathway, thereby inhibiting lipid accumulation, or it can bind to HSP90AA1 to modulate NF-κB signaling and alleviate inflammation (An et al., 2025). Meanwhile, the zwitterionic nature of BET optimizes drug delivery systems through its superhydrophilicity and resistance to nonspecific interfacial adsorption. For instance, polyalkyl BET (PCB) can stabilize liposomes, prolong their circulation half-life, and reduce immunogenicity (De Breuck et al., 2025), while cysteine BET-modified nanoshells achieve precise targeting while resisting biofouling. These properties effectively enhance pharmacokinetic profiles and targeting efficacy. Integrating current knowledge, a correlation appears to exist between ring system complexity and multi-target regulatory capacity: tetracyclic scaffolds, common in certain alkaloids, frequently act on multiple key nodes simultaneously, such as AMPK, NF-κB, and NLRP3, whereas structurally simpler pyridine- or indole-based alkaloids, such as koumine, exhibit relatively limited pathway selectivity. However, exceptions exist. Indole alkaloids, by virtue of their immunomodulatory activities, can ameliorate MASLD by influencing the Th1/Th2/Th17/Treg balance and the inflammatory cytokine network, indicating that even simple scaffolds can exert pleiotropic effects via specific mechanisms (Yue et al., 2019). Therefore, the relationship between ring system complexity and multi-target potential is not strictly linear and must account for pharmacophore distribution, substituent properties, and binding site affinity.

To sum up, most alkaloids that contain polycyclic aromatic structures along with suitable polar substituents typically aim to interact with key energy-sensing proteins like AMPK and PPARs. Looking ahead, the design of novel derivatives with improved pharmacokinetic properties and reduced off-target interactions may be advanced by SAR-guided modifications, such as ring cleavage, halogen addition, formation of dimers, or fine-tuning of hydrophobic moieties. As previously documented, the hydrophobic moieties attached to the pyridine ring play a pivotal role in determining the biological activity of sesquiterpene pyridine alkaloids. Additionally, the C-1 substituent and C-ring stereochemistry of β-cyclol alkaloids have been shown to exert a profound influence on their ability to inhibit lipid droplets, underscoring these structural features as key regions for fine-tuning both efficacy and target specificity (Long et al., 2021). Therefore, carrying out systematic structural modifications, along with thorough *in vivo* and *in vitro* assessments, will serve as a vital guide for developing the next-generation of MASLD therapeutic agents.

### 4.6 Structural optimization strategies for alkaloids and drug-drug interaction (DDI) risks

Natural product alkaloids often exhibit limitations such as weak pharmacological activity, poor aqueous solubility, and metabolic instability, necessitating structural modifications to enhance their drug-likeness. For instance, the antitumor activity of evodiamine was significantly enhanced following tetracyclic structural modification and optimization of its delivery system; furthermore, modifications to the A-ring substituents of steroidal alkaloids can augment their anticancer potency (Wang Z. et al., 2023). Concurrently, during the optimization process, careful consideration must be given to the potential inhibitory effects of alkaloids on metabolic enzymes and drug transporters. Alkaloids containing a methylenedioxyphenyl (MDP) group, such as BER, can cause time-dependent inhibition (TDI) of CYP3A4 (Mao, 2026). NUC can induce potential drug-drug interactions (DDIs) by inhibiting key CYP enzymes, notably CYP2D6 and CYP1A2, and it concurrently serves as a substrate for multiple CYP isoforms; however, relevant clinical data remain insufficient and warrant systematic evaluation (Liu Y. L. et al., 2026). Furthermore, compounds such as dihydroBER and tetrahydropalmatine exhibit over 50% inhibition of the liver-specific uptake transporter OATP1B1.

This membrane-bound protein plays a key role in the uptake of numerous pharmaceuticals and natural compounds into the liver from the bloodstream. While its ability to curb the buildup of harmful substances in the liver can be beneficial, it also gives rise to worries about possible disruptions to regular metabolic processes and a heightened susceptibility to drug-induced liver damage (Sun Y. H. et al., 2025). P-glycoprotein (P-gp) functions as an ATP-dependent drug efflux transporter. Within normal bodily tissues, including the intestines, liver, kidneys, and blood-brain barrier, it plays a key role in expelling foreign substances from cells, which in turn influences drug absorption, distribution, and elimination. In tumor cells, the overexpression of P-gp triggers the excessive efflux of chemotherapeutic drugs, leading to multidrug resistance (MDR). Conversely, dibenzylisoquinoline alkaloids suppress P-gp through their 18-membered ring configurations, showing potential to reverse MDR (Xu et al., 2020). Therefore, the future research and development of anti-MASLD alkaloid agents, along with their corresponding structural optimization, must carefully balance the enhancement of pharmacokinetic properties against the avoidance of CYP- and transporter-mediated DDI risks.

## 5 Integrated application of multi-omics technologies in MASLD research and translational insights

In recent years, the application of multi-omics technologies in MASLD research has shifted from single-modality analysis to spatially resolved and cross-tissue integrative paradigms. In the realm of spatial multi-omics, a study published in *Nature Genetics* constructed the first human spatial multi-omics atlas of MASLD (61 samples), integrating single-cell transcriptomics, spatial transcriptomics, and mass spectrometry imaging-based metabolomics. This study identified microphthalmia-associated transcription factor (MITF) as a key regulator of the lipid-processing ability of lipid-associated macrophages (LAMs), and discovered that LAMs exert their hepatoprotective effects by secreting hepatocyte growth factor (HGF) (Li Z. Y. et al., 2025). Regarding cross-kingdom multi-omics of the gut microbiome, a large-sample study (211 MASLD cases/502 healthy controls) integrating metagenomics, metatranscriptomics, and metabolomics found that MASLD was significantly associated with alterations in the abundance of 66 bacterial species, enrichment of oral microbes, and perturbations in the virome. These changes were accompanied by elevated polyamine and acylcarnitine levels, alongside reduced secondary bile acid levels (Kim et al., 2025). Furthermore, a pediatric MASLD study employing 16S rDNA sequencing combined with targeted metabolomics revealed a positive correlation between the abundance of *Ruminococcus torques* and both deoxycholate levels and liver stiffness values, suggesting the potential of this bacterial strain as a novel target for disease management (Du et al., 2025).

Multi-omics technologies are now deeply integrated into MASLD research, with their application evolving from descriptive analysis to mechanistic validation and precision intervention. Metabolomics, through untargeted and targeted analyses, has identified serum metabolic biomarkers (e.g., TG and sphingomyelins) that distinguish NASH from NASH-HCC, and has revealed significant reprogramming of programmed necrosis and lipid metabolic pathways during disease progression (Ahmed et al., 2022). Proteomics, via quantitative analysis, has uncovered the dysregulation of key proteins, such as MTTP and CYP2C6, in lipid transport and xenobiotic metabolism, and has identified core targets and novel biomarkers through network analysis (Abo Nahas et al., 2026). In natural product research, multi-omics approaches are also widely utilized for mechanistic elucidation; for example, demonstrating that ginsenoside Rg1 regulates tryptophan metabolism in an intestinal microbiota-dependent manner, and that naringenin modulates PI3K-AKT signaling and triglyceride homeostasis (Sun W. P. et al., 2025). These strategies share multiple points of convergence with the exploration of bioavailability: metabolomics can monitor the *in vivo* dynamics of a drug and its metabolites to assess ADME properties, and indirectly predict transporter or enzyme activity by leveraging changes in endogenous metabolites under disease conditions. Proteomics can assess the impact on absorption and first-pass metabolism by analyzing changes in the expression of CYP450 enzymes and transporters in the gut and liver under MASLD conditions; when combined with lipidomics to analyze microenvironmental alterations, this approach can predict drug distribution and metabolic rates. Furthermore, the latest machine learning models can guide dose optimization based on individual metabolic profiles by stratifying fibrosis and NAS scores, thereby enhancing bioavailability (Xu et al., 2026). In summary, by elucidating gut microbial metabolism, host target responses, and drug tissue distribution, multi-omics approaches comprehensively clarify the causal relationships between actual compound exposure and pharmacodynamic effects. This provides a systematic basis for optimizing dosing strategies and developing novel drugs with high bioavailability in the context of MASLD.

## 6 Conclusion

Metabolic dysfunction-associated steatotic liver disease (MASLD) has emerged as one of the most prevalent chronic liver diseases globally. Its intricate pathogenesis encompasses a continuum of pathophysiological events, including lipid metabolic dysregulation, IR, gut microbiota dysbiosis, aberrant cell death modalities, and severe inflammatory responses. Natural plant-derived alkaloids have become a focal point in novel drug discovery owing to their multi-target engagement, broad-spectrum pathway modulation, high target selectivity, robust biological activity, and favorable safety profiles. The therapeutic application of natural products is rapidly transitioning from a traditional empirical paradigm to a rigorous, science-driven discipline with exceptional translational potential. This review has comprehensively synthesized the therapeutic potential and corresponding molecular targets of diverse natural bioactive alkaloids in the context of MASLD. Through the activation of AMPK, the upregulation of PPARα/CPT1 to accelerate fatty acid oxidation, and the downregulation of SREBP-1c/ACC/PPARγ to halt lipogenesis, these alkaloids profoundly restore gut-liver axis function, neutralize inflammatory cascades, and mitigate hepatocellular death. By detailing how these alkaloids alleviate metabolic perturbations and hepatic injury, this review delineates novel avenues for innovative drug development. These insights will facilitate the discovery of novel MASLD biomarkers and the clinical translation of advanced therapeutics. In conclusion, natural alkaloids hold immense prophylactic and therapeutic promise in the global fight against MASLD.

## 7 Future direction

Alkaloids from natural products play a distinct role in addressing MASLD, as their key strengths lie in their inherent structural complexity and ability to target multiple interconnected pathological pathways. Their polycyclic scaffolds, such as the isoquinoline ring in BER, the aporphine skeleton in NUC, and the zwitterionic group in BET, can simultaneously modulate multiple interrelated pathological nodes, including AMPK energy sensing, NLRP3 inflammasome inhibition, GPX4-mediated ferroptosis defense, and Tj repair. This multi-target engagement precisely addresses the complex pathological network outlined by the current multiple-hit hypothesis of MASLD. Encouragingly, this mechanistic framework is increasingly supported by evidence from translational medicine. Notably, emerging findings from clinical research further validate this mechanistic model. For instance, Phase II/III clinical imaging studies have confirmed that BER significantly and reproducibly alleviates hepatic steatosis. Similarly, preclinical studies have demonstrated that NUC and BET improve multiple endpoint indicators, including lipid deposition, inflammation, and fibrosis.

Although natural product alkaloids hold great promise for MASLD therapy, their clinical application is hindered by three core bottlenecks: ambiguous molecular targets, poor oral bioavailability, and a significant gap between animal efficacy and clinical translation. The mechanisms of action for most alkaloids remain confined to descriptive characterizations of downstream signaling pathways, while their direct protein targets remain elusive. This impedes the establishment of SARs and the optimization of target selectivity. Future research should leverage chemical proteomics and biophysical approaches to precisely identify the direct protein targets of these alkaloids. Based on newly identified scaffolds and well-established pathways such as PPARα, PI3K/AKT, and AMPK/Nrf2, systematic SAR studies should be conducted to minimize off-target effects. Furthermore, integrating single-cell transcriptomics, spatial transcriptomics, and lipidomics will facilitate the dissection—at single-cell resolution—of the differential effects of alkaloids on various hepatic cell subpopulations, as well as their reprogramming effects on pathological sphingolipid metabolism (e.g., ceramides). Concurrently, implementing 3D human hepatocyte spheroids and liver-biliary organoid models will help predict human-specific metabolism and toxicity, thereby bridging the interspecies translational gap.

In terms of pharmacokinetics, certain alkaloids exhibit remarkably low systemic exposure due to poor aqueous solubility, limited intestinal permeability, and strong first-pass metabolism. Nevertheless, substantial efficacy has been consistently observed in animal models, suggesting that these compounds may exert their effects indirectly via local intestinal or microbiota-mediated pathways rather than depending on systemic concentrations of the parent drug, thereby presenting a “pharmacokinetic-pharmacodynamic paradox.” Consequently, the optimization of alkaloid formulations should not solely aim to increase overall systemic exposure; rather, it should prioritize the identification of primary target sites prior to targeted formulation enhancement. A combinatorial strategy employing lecithin-based phospholipid complexes alongside piperine could be implemented (Tawfik et al., 2026). The former enhances lipophilicity, whereas the latter inhibits metabolism, thereby collectively augmenting the absorption of the active compound. Alternatively, advanced nanotechnology can be leveraged to incorporate these natural products into nano-delivery systems (Egba et al., 2025). However, the applicability and safety of these approaches in the context of MASLD warrant further validation.

Currently, most existing clinical trials on MASLD treatment are limited by small sample sizes, short study periods, and a lack of liver biopsies, which serve as the diagnostic gold standard for evaluating disease stages. This limitation hinders accurate assessment of the true therapeutic effects and safety profiles of potential interventions across different disease stages. Currently, BER remains one of the few alkaloids validated by RCTs. A double-blind, placebo-controlled trial involving 70 overweight and mildly obese patients with MASLD demonstrated that a 12-week intervention with 1,500 mg/day of BER was safe and well-tolerated, with only a few subjects experiencing mild, transient gastrointestinal adverse events (Koperska et al., 2024); however, high doses may precipitate adverse effects such as cardiac suppression. To date, no RCTs have systematically evaluated the efficacy and safety of NUC in MASLD. Regarding BET, only two observational studies are currently available (Omileke et al., 2025): one suggested an inverse association between dietary BET intake and the risk of overweight/obesity (n = 492), while the other identified a correlation between plasma BET levels and brain structural alterations in patients with schizophrenia (n = 76). These observational studies merely suggest correlations without establishing causality, underscoring a clear insufficiency in the current level of evidence.

To bridge the gap in translational efficacy, future research should conduct dose-escalation studies guided by systemic pharmacokinetic/pharmacodynamic (PK/PD) modeling, establish pathology-specific dose-adjustment models, and integrate the gut-liver axis and bile acid metabolic pathways to comprehensively evaluate the impact of transport proteins on drug exposure. Furthermore, considering the limitations of conventional animal models in fully simulating human physiological diversity, it is necessary to adopt advanced platforms like humanized mice and organ-on-a-chip systems (Mehra et al., 2026). Regarding safety evaluations, foundational toxicological research on these alkaloids is severely lagging. Priority should be given to conducting sub-chronic and chronic toxicity assays, with a specific focus on adverse effects within the hepatic, renal, and biliary systems, utilizing metabolomics to identify robust toxicity biomarkers. To facilitate clinical application, strict criteria comparable to those for synthetic pharmaceuticals are necessary, including well-defined chemical formulations, batch-to-batch reproducibility, and the development of reliable quality control (QC) protocols. Moreover, studies should integrate systematic PK/PD approaches to clarify how target organ exposure translates into therapeutic outcomes, and regulatory standards for long-term safety assessments must remain rigorous regardless of whether the compounds are derived from natural sources. The design of clinical trials should be structured in phases, prioritizing improvements in liver histology as the key outcome. This approach must also include larger study populations, longer periods of follow-up, and thorough assessments of how multiple medications interact when patients have additional health issues. Regulatory requirements for long-term safety evaluations should not be relaxed simply because these compounds are of “natural” origin. Given the highly overlapping pathological mechanisms among metabolic disorders, it is imperative to further investigate whether these bioactive natural products can simultaneously mitigate multiple metabolic-related conditions, such as cardiovascular and cerebrovascular diseases.

To advance early prevention, accurate diagnosis, and sustained remission of MASLD, future studies need to proactively investigate personalized therapeutic approaches. Notably, host genetic backgrounds, particularly the *PNPLA3* I148M variant, may significantly influence the therapeutic efficacy of alkaloids. The *PNPLA3* I148M variant predisposes hepatocytes to an inherent state of lipid metabolic dysregulation, characterized by attenuated triglyceride hydrolytic activity and impaired protein degradation (Das et al., 2024). This in turn may result in disruptions to Nrf2-dependent antioxidant mechanisms and mitochondrial function, which are exactly the primary pathways that alkaloids like BER, NUC, and BET target through the AMPK/PPARα/GPX4 axis. Individuals carrying this risk allele could show increased susceptibility to these alkaloids or display markedly different therapeutic outcomes. Future studies should stratify animal models by *PNPLA3* genotypes *a priori* to systematically evaluate the differential interventional effects of representative alkaloids on hepatic steatosis, inflammation, and fibrosis, thereby establishing a foundation for genotype-guided precision therapies using natural products.

In conclusion, the research and development of natural product alkaloids should be incorporated into the framework of evidence-based medicine. Only through high-quality clinical trials to verify their prognostic value and safety can MASLD be transformed from an obscure epidemic into a predictable, preventable and precisely controllable chronic disease. We hope this review can offer a direction for the development of efficacious and highly bioavailable natural products.
